# SENP3-mediated host defense response contains HBV replication and restores protein synthesis

**DOI:** 10.1371/journal.pone.0209179

**Published:** 2019-01-14

**Authors:** Rui Xi, Preetish Kadur Lakshminarasimha Murthy, Kuei-Ling Tung, Cynthia D. Guy, Ji Wan, Feng Li, Zhuo Wang, Xiaodong Li, Anastasia Varanko, Nikolai Rakhilin, Yongning Xin, Botao Liu, Shu-Bing Qian, Lishan Su, Yan Han, Xiling Shen

**Affiliations:** 1 Department of Biomedical Engineering, Pratt School of Engineering, Duke University, Durham, North Carolina, United States of America; 2 Duke Center for Genomic and Computational Biology, Duke University, Durham, North Carolina, United States of America; 3 Duke Cancer Institute, Duke University, Durham, North Carolina, United States of America; 4 Sibley School of Mechanical and Aerospace Engineering, Cornell University, Ithaca, New York, United States of America; 5 Department of Biological and Environmental Engineering, Cornell University, Ithaca, New York, United States of America; 6 Department of Pathology, Duke University School of Medicine, Durham, North Carolina, United States of America; 7 Division of Nutritional Science, College of Human Ecology, Cornell University, Ithaca, New York, United States of America; 8 Lineberger Comprehensive Cancer Center, School of Medicine, University of North Carolina at Chapel Hill, Chapel Hill, North Carolina, United States of America; 9 Institute of Infectious Diseases, Guangzhou Eighth People’s Hospital, Guangzhou, China; 10 School of Electrical and Computer Engineering, College of Engineering, Cornell University, Ithaca, New York, United States of America; 11 Department of Gastroenterology, Qingdao Municipal Hospital, Qingdao, China; 12 Digestive Disease Key Laboratory of Qingdao, Qingdao, China; 13 Medical College of Qingdao University, Qingdao, China; 14 Program in Molecular Medicine, University of Massachusetts Medical School, Worcester, Massachusetts, United States of America; Indiana University, UNITED STATES

## Abstract

Certain organs are capable of containing the replication of various types of viruses. In the liver, infection of Hepatitis B virus (HBV), the etiological factor of Hepatitis B and hepatocellular carcinoma (HCC), often remains asymptomatic and leads to a chronic carrier state. Here we investigated how hepatocytes contain HBV replication and promote their own survival by orchestrating a translational defense mechanism via the stress-sensitive SUMO-2/3-specific peptidase SENP3. We found that SENP3 expression level decreased in HBV-infected hepatocytes in various models including HepG2-NTCP cell lines and a humanized mouse model. Downregulation of SENP3 reduced HBV replication and boosted host protein translation. We also discovered that IQGAP2, a Ras GTPase-activating-like protein, is a key substrate for SENP3-mediated de-SUMOylation. Downregulation of SENP3 in HBV infected cells facilitated IQGAP2 SUMOylation and degradation, which leads to suppression of HBV gene expression and restoration of global translation of host genes via modulation of AKT phosphorylation. Thus, The SENP3-IQGAP2 de-SUMOylation axis is a host defense mechanism of hepatocytes that restores host protein translation and suppresses HBV gene expression.

## Introduction

Hepatitis B virus (HBV) causes hepatitis B, a liver infectious disease affecting a significant population of people worldwide[1]. It is also a predominant etiological factor for hepatocellular carcinoma (HCC)[2, 3]. The immune system is often credited with containment and clearance of HBV[4]; nevertheless, infected hepatocytes produce varying amounts of HBV virus, estimated to be as low as 1–10 virions per day by some reports[5, 6]. Host factors such as the SMC5/6 complex have been identified to restrict HBV transcription, but HBV regulatory protein X (HBx) targets the complex for degradation, hence removing the obstacle[7, 8]. It is unclear whether hepatocytes are capable of mounting an intrinsic defense mechanism to contain viral replication.

SUMOylation is a highly dynamic post-translational modification process reversible by SUMO-specific peptidases (SENPs), a family of proteases that catalyze de-conjugation of SUMO proteins (de-SUMOylation) [9, 10]. Both SUMOylation and de-SUMOylation play important roles in cellular processes such as DNA replication and repair, cell division, apoptosis, cancer and stress responses[11–15]. SENP enzymes maintain the balance between SUMOylation and de-SUMOylation. SENP3, one of the six SENP isoforms, has the specificity for SUMO-2/3 deconjugation on target proteins, although only a few targets have been identified[16]. It has been reported that SENP3 acts as a sensor for a variety of stresses, such as reactive oxygen species (ROS), hypoxia, and bacterial infection[17]. SENP3 is a dual-faceted regulator of cell survival and growth, enhancing cell proliferation under low level of ROS while promoting cell apoptosis under high level of ROS[18, 19].

In this study, we report a SENP3-IQGAP2 SUMOylation axis in hepatocytes that restores global host translational activity and contains HBV gene expression in response to HBV infection. Upon HBV infection, hepatocytes downregulate SENP3 to SUMOylate and degrade IQGAP2, a Ras GTPase-activating-like protein we identified as a new SENP3 substrate. Degradation of IQGAP2 relieves its suppression of Akt phosphorylation, which promotes host translation and suppresses HBV protein expression. Thus, the SENP3-IQGAP2 SUMOylation axis acts as a host defense mechanism to reboot host gene translation for cell survival.

## Materials and methods

### Ethics statement

Formalin-fixed, paraffin-embedded (FFPE) sections of HBV-infected human liver tissue were acquired from the Duke Translational Research Institute Biobank (BRPC-15-876). The FFPE sections of normal human liver tissue are acquired from Dr. Cynthia Guy’s Liver Lesions Database in the Duke Department of Pathology. The approval from the Duke Health Institutional Review Board to use the samples was received. All samples were anonymized.

### Cell culture

Human hepatic cancer cell line HepG2 and human embryonic kidney 293T (HEK293T)-derived 293T cell line were purchased from ATCC. HepG2-derived HBV-producing stable cell line HepG2.215 was provided by Dr. Andrea Cuconati. HepG2-NTCP cells expressing the HBV receptor sodium taurocholate cotransporting polypeptide (NTCP), HepG2-HBx cells expressing HBx upon doxycycline induction, and HepAD38 cells producing HBV under the control of a tetracycline inducible promoter, have been previously reported[8, 20]. All the cell lines were cultured in Dulbecco’s modified Eagle’s medium (DMEM) supplemented with 10% fetal bovine serum (FBS) and L-glutamine (2 mM). Cells were incubated at 37 °C in 5% CO_2_.

### Short-hairpin RNA (shRNA) gene silencing

Predesigned sequence-specific MISSION shRNA vectors, pLKO.1-puro (control) vector, lentiviral packaging vectors were purchased from SIGMA-ALDRICH in the form of bacterial glycerol stock. The sequences of MISSION shRNAs are listed in S1A Table. Plasmids were extracted using QIAGEN Plasmid Maxi Kit. HEK293T cells were transfected with the plasmids to package lentiviruses using TransIT-LT1 Transfection Reagent per instructions in the manual. The collected lentiviruses were used to infect the described cell lines to silence or mock silence the genes of interest. Puromycin (1 μg/ml, Thermo Fisher Scientific) was added to the cell culture medium for selection. The efficiency of gene silencing was verified by both RT-qPCR and immunoblotting.

### Western blotting/immunoblotting

Cellular lysate was subjected to a standard Bio-Rad western blotting workflow using Mini-PROTEAN TGX Stain-Free Precast Gels and Trans-Blot Turbo Transfer System. The primary antibodies used were listed in S1B Table. The corresponding secondary antibodies were purchased from Santa Cruz Biotechnology. Beta-actin was used as a loading control. Protein bands were processed using Pierce ECL Western Blotting Substrate (Thermo Fisher Scientific) followed by visualization in a ChemiDoc Touch Imaging System (Bio-Rad). Images were edited in Image Lab Software (Bio-Rad).

### Real-time Reverse-Transcription Quantitative PCR (RT-qPCR)

Total RNA was extracted using TRIzol Reagent (Thermo Fisher Scientific) per instructions in the manual. cDNA was synthesized using QuantiTect Reverse Transcription Kit (QIAGEN). PCR reactions were prepared using QuantiFast SYBR Green PCR Kit (QIAGEN). Real-time RT-PCR was performed using an Applied Biosystems StepOnePlus Real-Time PCR System in a two-step cycling protocol, with a denaturation step at 95 °C and a combined annealing/extension step at 60 °C. The primers (S1C Table) were purchased from Integrated DNA Technologies.

### Immunofluorescent (IF) staining

Slides were deparaffinised using xylene and rehydrated. Heat induced antigen retrieval was performed using Tris-based buffer (Vector Labs). Sections were then blocked using Serum-free protein block (Dako) and incubated with primary antibody (listed in S1B Table) overnight at 40C. Slides were washed in PBS and incubated with fluorescent-tagged (Abcam) for one hour respectively at room temperature. Slides incubated with fluorescent antibodies were washed, treated with TrueBlack (Biotium) to reduce autofluorescence and then mounted using Vectashield medium with DAPI (Vector Labs).

### HBV collection, infection and detection

HepAD38 cells were grown for 7 days. Media was collected and centrifuged at 10,000 g. 10% polyethylene glycol (PEG)-8000 was added to the supernatants, which were incubated at 21°C for 30min and 4°C for 60min, followed by centrifugation. The pellet was re-suspended in serum-free media in 1/100 the original volume to make HBV stocks. Before HBV infection, HepG2-NTCP cells (control and SENP3 K.D.) were plated at 70%-80% confluency and were infected by the collected HBV at an MOI of 200 in serum free DMEM media with 4% PEG-8000. After 16 hr, the inoculum was removed and the infected cells were rinsed three times with PBS. Complete DMEM media with 2% DMSO was added to culture the cells and collected every 24 hr. Infected HepG2-NTCPs cells were collected for RNA extraction. To measure viral load, HBV genome DNA in supernatants was extracted using QIAamp MinElute Virus Spin Kit (QIAGEN). The DNA was subjected to qPCR amplified by primers listed in S1C Table. HBV 1.3-mer WT replicon plasmid was used as the control for calculating HBV genome copy number.

### Transient transfection

The pcDNA3.1 vector was used as control construct. SENP3 was cloned into pcDNA3.1 vector for FLAG-tagging. The RGS-His (RH)-tagged SENP3 constructs and the RGH-His-tagged SENP3 mutant construct were purchased from Addgene. Cells were plated in six well plates at 80–90% confluency 24 hrs before transfection. TransIT-LT1 transfection reagent was used for transfection of plasmids according to the manual. Cells were harvested after 24–48 hrs.

### Drug treatment

Cells were treated with MG132 (working concentration: 1 μM), general mTOR inhibitor Rapamycin (working concentration: 20 nM), PI3K inhibitor LY294002 (working concentration: 20 μM) for 6 hrs. For labeling, puromycin (working concentration: 100 μg/mL) treated the cells for 30 min. For induction of HBx expression, HepG2-HBx cells were treated with doxycycline (500 ng/ml) for 5 days.

### Immunoprecipitation (IP)

Cells were collected with RIPA buffer (Sigma) supplemented with EDTA-free cocktail protease inhibitor (Roche) and 1mM N-Ethylmaleimide. The whole cell lysates were incubated with antibodies incubated at 4°C for 1 hr. 30 μL Pierce Protein A/G magnetic beads (Thermo Scientific) were added to the mixture and incubated at room temperature for 1 hour. The beads were carefully washed three times with RIPA buffer, followed by elution with 60 mM Tris-HCL, PH 6.8, 25% Glycerol, 2% SDS, 14.4 mM β-Mercapitoethanol, 0.1% Bromophenol Blue.

### Protein mass spectrometry work flow

Following IP, the protein samples were run through an SDS-PAGE gel. The bands were excised into small pieces and subjected to in-gel trypsin digestion for extracting the tryptic peptide as described previously[21]. Nano liquid chromatography tandem mass spectrometry (nano-LC-ESI MS/MS) was performed on the in-gel tryptic digests by Proteomics & Mass Spectrometry Facility using an Orbitrap Fusion Tribrid mass spectrometer equipped with a Nanospray Flex Ion Source (Thermo Fisher Scientific) along with an UltiMate 3000 RSLCnano System (Thermo Scientific). The data were obtained using Xcalibur Software (Thermo-Fisher Scientific). Proteome Discoverer 1.4 Software (PD1.4, Thermo Fisher Scientific) was used for outputting all MS and MS/MS raw spectra in MGF format followed by protein identification by Mascot search engine (Matrix Science). Database searches were performed using a human RefSeq sequence database with a setting of 2 missed cleavage sites by trypsin, 10ppm peptide tolerance and 0.6 Da MS/MS tolerance. Variable modifications that were set included carbamidomethylation of cysteine, deamidation and methionine oxidation of asparagines/glutamine residues. Data filtering parameters included (i) less than 1% false discovery rate (FDR); (b) probability of the peptide ID is 95% confidence interval (CI) with peptide expect cutoff at 0.05.

### Sucrose cushion

10^6^ cells were lysed in 300 μL polysome lysis buffer (10 mM HEPES, pH 7.4, 100 mM KCl, 5 mM MgCl2, 5 mM DTT, EDTA-free protease inhibitor cocktail, 100 ug/mL CHX, 2% TritonX-100) on ice for 10min. 50 μL of the lysate was used for RNA-seq. 7.5 μL RNase I (100U/uL) were added to the remaining lysates and rotated at 4°C for 1 hr. 10 μL SUPERase-In (Ambion) was added to terminate nuclease digestion. 250 μL digested lysate was loaded on top of 0.9 mL 1M sucrose prepared in modified polysome buffer (10 mM HEPES, pH 7.4, 100 mM KCl, 5 mM MgCl2, 5 mM DTT, EDTA-free protease inhibitor cocktail,) in a 5mL ultrocentrifuge tube. After centrifugation at 90,000 rpm for 160 min at 4°C using a Beckman TLA-110 rotor, the pellet was collected in 1 mL TRIzol reagent (Thermo Fisher Scientific).

### Ribosome-Protected Fragments (RPFs) deep sequencing library

RPF deep sequencing library was created according to a previous work[22]. In brief, following the sucrose cushion, the RPFs were purified by TRIzol reagent according to the manual. Purified RPFs were dephosphorylated with T4 polynucleotide kinase (New England Biolabs) for 1 hr at 37 °C, then heat inactivated at 65 °C for 20 min. Dephosphorylated samples were purified with a Novex denaturing 15% polyacrylamide TBE-urea gel (Invitrogen). RNA fragments around 28 nt were recovered overnight in gel elution buffer (300 mM NaOAc, pH 5.5, 1 mM EDTA, 0.1 U/ml SUPERase_In). Purified RNA fragments were processed by Poly-(A) tailing reaction with E. coli poly-(A) polymerase (NEB) followed by reverse transcription with the following oligos:

5-pCAGATCGTCGGACTGTAGAACTCT∅CAAGCAGAAGACGGCATACGATTTTTTTTTTTTTTTTTTTTVN-3;

5-pGTGATCGTCGGACTGTAGAACTCT∅CAAGCAGAAGACGGCATACGATTTTTTTTTTTTTTTTTTTTVN-3;

5-pTCGATCGTCGGACTGTAGAACTCT∅CAAGCAGAAGACGGCATACGATTTTTTTTTTTTTTTTTTTTVN-3;

Reverse transcription reaction was performed with SuperScript III (Invitrogen) per the manufacturer’s instructions. Reverse transcription products were purified on a 10% polyacrylamide TBE-urea gel and recovered by DNA gel elution buffer (300 mM NaCl, 1 mM EDTA). The purified cDNA was circularized with CircLigase II (Epicentre) according to manufacturer’s instructions. The circulated products were purified with isopropanol. The circulated template was amplified by PCR with the Phusion High-Fidelity enzyme (NEB) per the manufacturer’s instructions. Primers (5′-CAAGCAGAAGACGGCATA-3′) and (5′AATGATACGGCGACCACCGACA GGTTCAGAGTTCTACAGTCCGACG-3′) were used to create the sequencing library. PCR products were purified with 8% polyacrylamide TBE gel and recovered with DNA gel elution buffer. The products were quantified by Agilent BioAnalyzer DNA 1000 assay; the pooled barcoded samples were sequenced by using sequencing primer 5′-CGACAGGTTCAGAGTTCTACAGTCCGACGATC-3′ (Illumina HiSeq 2500).

### Ribosome profiling analysis

The raw reads (50nt) of ribosome profiling were preprocessed by removing 5’ barcode (2nt) and 3’ polyA tail (allowing one mismatch). The processed reads with lengths ranging from 25nt to 35nt were retained for downstream analyses. Tophat and Bowtie[23, 24] were used to map processed reads to human or HBV transcriptome and genome with default parameters. Reads with only one mapping location were extracted using SAMtools (samtools view –bq 20)[25]. To avoid ambiguity in interpreting ribosome profiling data in the context of mRNA splicing isoforms, a set of longest mRNA transcripts were compiled by comparing different mRNA isoforms of the same gene on CDS length (if CDS lengths are the same, 5’ UTR and 3’ UTR are compared respectively). To dissect the ribosome footprint in a nucleotide resolution, the 13th position (12nt offset from the 5’ end) of the uniquely mapped read to mRNA was defined as the “P-site” during protein synthesis. P-sites located in the mRNA CDS were summarized to calculate the normalized ribosome footprint densities of individual mRNAs using the following formula:

$${Ribosome\;density}_{norm}=\frac{{P-site\;reads}_{CDS}\times 10^{9}}{Total\;mapped\;reads\times CDS\;length}$$

The raw reads of mRNA sequencing were preprocessed in the same way as the ribosome profiling. The processed reads greater than 20nt were retained for gene abundance estimation using tophat and Cufflinks. To make pair-wise comparison of mRNA translation efficiency, the normalized ribosome density was further normalized by Cufflinks-derived FPKM value of the corresponding gene.

### Statistical analysis

Comparison of two groups of data were performed using unpaired two-tailed Student’s t test. P-value < 0.05 was considered the threshold for statistical significance. Data were expressed as mean±SD.

### Access to data

All authors have access to the study data and have reviewed and approved the final manuscript.

## Results

### SENP3 is downregulated in HBV-infected hepatocytes

**Fig 1:**
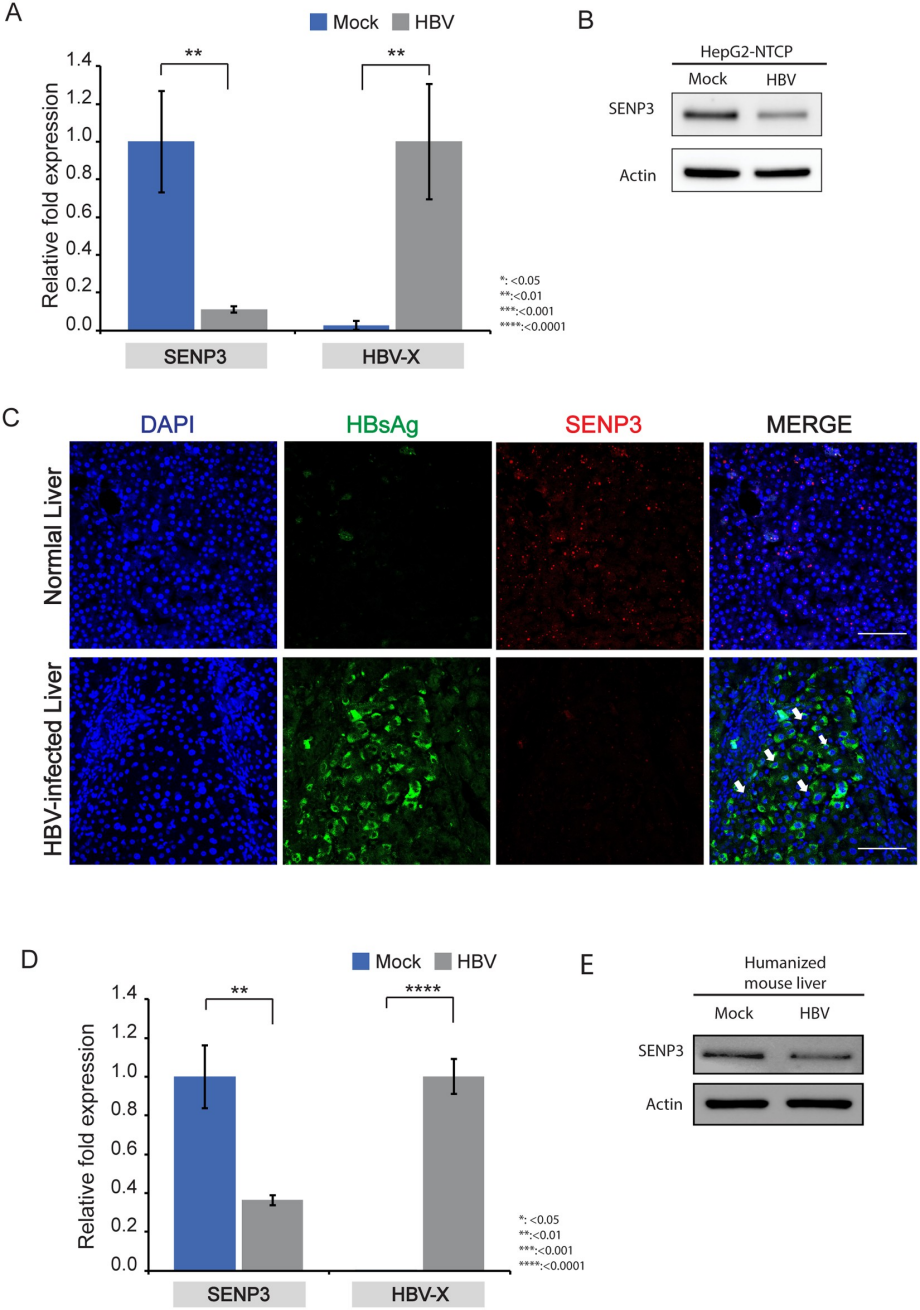
SENP3 is downregulated in HBV-infected hepatocytes. (A) RT-qPCR measurement of SENP3 mRNA level in HepG2-NTCP cells on Day 7 after HBV infection and mock infection. HBV transcripts were amplified by the primer pair HBV-X to indicate the presence of HBV in the cells. Beta-actin was used as internal control; the data were expressed as mean±SD (n = 3). The statistical significance was assessed by Students’ unpaired *t*-test. (B) Immunoblotting SENP3 steady-state level in HepG2-NTCP cells on Day 7 after HBV infection and mock infection. (C) Representative immunofluorescence (IF) images of SENP3 protein levels in tissue sections of normal and HBV-infected livers. The white arrow indicates SENP3 signal. Scale bar indicates 100 μm. (D) RT-qPCR measurement of SENP3 mRNA level (n = 3) in HBV-infected and uninfected livers from a humanized mouse model (A2/NSGNRF-FAH-huHSC/Hep). Beta-actin was used as internal control; the data were expressed as mean±SD (n = 3). The statistical significance was assessed by Students’ unpaired *t*-test. (E) Immunoblotting of SENP3 translational level in HBV-infected and uninfected livers from a humanized mouse model (A2/NSGNRF-FAH-huHSC/Hep).

To explore the role of SENP3 in HBV-infected hepatocytes, we first examined SENP3 expression in HBV-infected HepG2-derived cells expressing the HBV receptor sodium taurocholate cotransporting polypeptide (HepG2-NTCP). RT-qPCR and immunoblotting showed that SENP3 decreased significantly in HepG2-NTCP cells after HBV infection compared with HepG2-NTCP cells that had mock infection as control (Fig 1A and 1B, S1 Fig). Immunofluorescence (IF) showed that expression of SENP3 was downregulated in HBV-infected human liver tissue compared with normal human liver tissue (Fig 1C, S2 Fig). We then examined SENP3 expression in livers of a humanized mouse model (A2/NSGNRF-FAH-huHSC/Hep) reconstituted with both human immune system and human liver cells, which enable persistent HBV infection[26–28]. RT-qPCR and immunoblotting confirmed the decrease of SENP3 expression in HBV-infected humanized liver (Fig 1D and 1E). SENP3 expression was lower in HepG2-derived HBV-producing stable cell line HepG2.215 than in HepG2 cells (S3 Fig). SENP3 expression level also decreased in HepG2-HBx cells that express FLAG-SBP-HBx encoding HBV regulatory X protein upon doxycycline induction (S4 Fig). Taken together, SENP3 was consistently found in different models to be downregulated in HBV-infected hepatocytes.

### SENP3 silencing suppresses HBV gene expression

**Fig 2:**
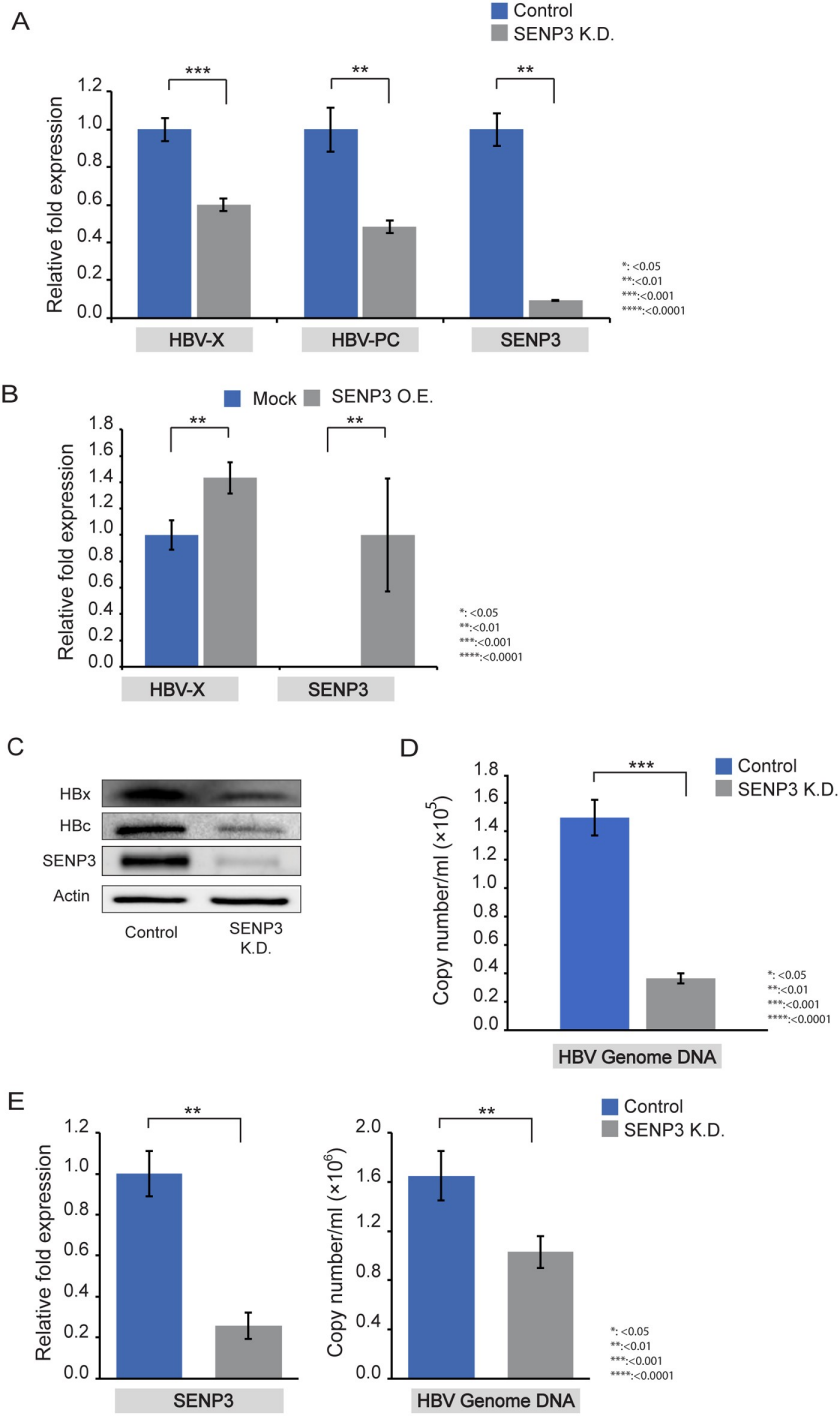
SENP3 silencing suppresses HBV gene expression. (A) RT-qPCR measurements of HBV transcripts amplified by primers HBV-PC and HBV-X in HepG2.215-control cells and HepG2.215-SENP3 K.D. cells. Beta-actin was used as internal control; the data were expressed as mean±SD (n = 3). The statistical significance was assessed by Students’ unpaired *t*-test. (B) RT-qPCR measurement of HBV transcripts amplified by primer pair HBV-X in HepG2.215-SENP3 K.D. cells with transient transfection of pcDNA3 plasmid (Mock) and RGS-SENP3 plasmid (SENP3 O.E.). Beta-actin was used as internal control; the data were expressed as mean±SD (n = 3). The statistical significance was assessed by Students’ unpaired *t*-test. (C) Immunoblotting of HBx and HBc in HepG2.215 control cells and HepG2.215 SENP3 K.D. cells. (D) qPCR measurements of HBV genome copy numbers in supernatants of HepG2.215 cells (control and SENP3 K.D.). HBV 1.3-mer WT replicon plasmid was used as internal control. The data were mean±SD (n = 3) and the statistical significance was assessed by Students’ unpaired *t*-test. (E) Left: RT-qPCR confirmation of SENP3 knockdown in HepG2-NTCP cells. Right: qPCR measurements of HBV genome copy numbers in supernatants of HepG2-NTCP cells (control and SENP3 K.D.) on Day 7 after HBV infection. Beta-actin (left) and HBV 1.3-mer WT replicon plasmid (right) were used as internal control. The data were mean±SD (n = 3) and the statistical significance was assessed by Students’ unpaired *t*-test.

To test whether downregulation of SENP3 had any impact on viral gene expression, we measured both transcriptional level of the HBV genome and representative HBV protein levels. The HBV genome is made of a partially double-stranded circular DNA, which is about 3.2 kilobase (kb) pairs long[29]. Upon entry into the nucleus, the viral genome undergoes a repair process and is circularized to the covalently closed circular (cccDNA) form. cccDNA encodes various overlapping transcripts that serve as templates of functionally important HBV proteins[30]. We used two pairs of RT-qPCR primers (HBV-PC and HBV-X) that have been established to amplify different regions of HBV genome to measure HBV transcriptional levels[31–33] (S2 Table). Pre-core mRNA levels can be detected by using HBV-PC qPCR primers, while HBV-X primers used here detect pgRNA, pre-core mRNA, X mRNA, and 2.4 and 2.1kb mRNAs. SENP3 knockdown significantly reduced viral transcription in HepG2.215 as well as in another HBV-producing cell line HepAD38 (Fig 2A, S5A Fig). HBV transcription in HepG2.215 cells with SENP3 knockdown can be restored by ectopic expression of wild-type SENP3, but not by an SENP3 mutant (C532A)[34] that lacks de-SUMOylating activity (Fig 2B, S5B Fig). Therefore, downregulation of SENP3 reduces HBV gene expression in a manner dependent on its de-SUMOylating activity. Levels of viral protein HBx and core protein HBc decreased after SENP3 knockdown in HepG2.215 cells (Fig 2C). SENP3 knockdown also reduced viral loads detected in the supernatants of HepG2.215 cells and HepG2-NTCP cells infected with HBV (Fig 2D and 2E, S5C Fig). This suggests that downregulation of SENP3 reduces HBV replication.

### SENP3 silencing restores host protein translation

**Fig 3:**
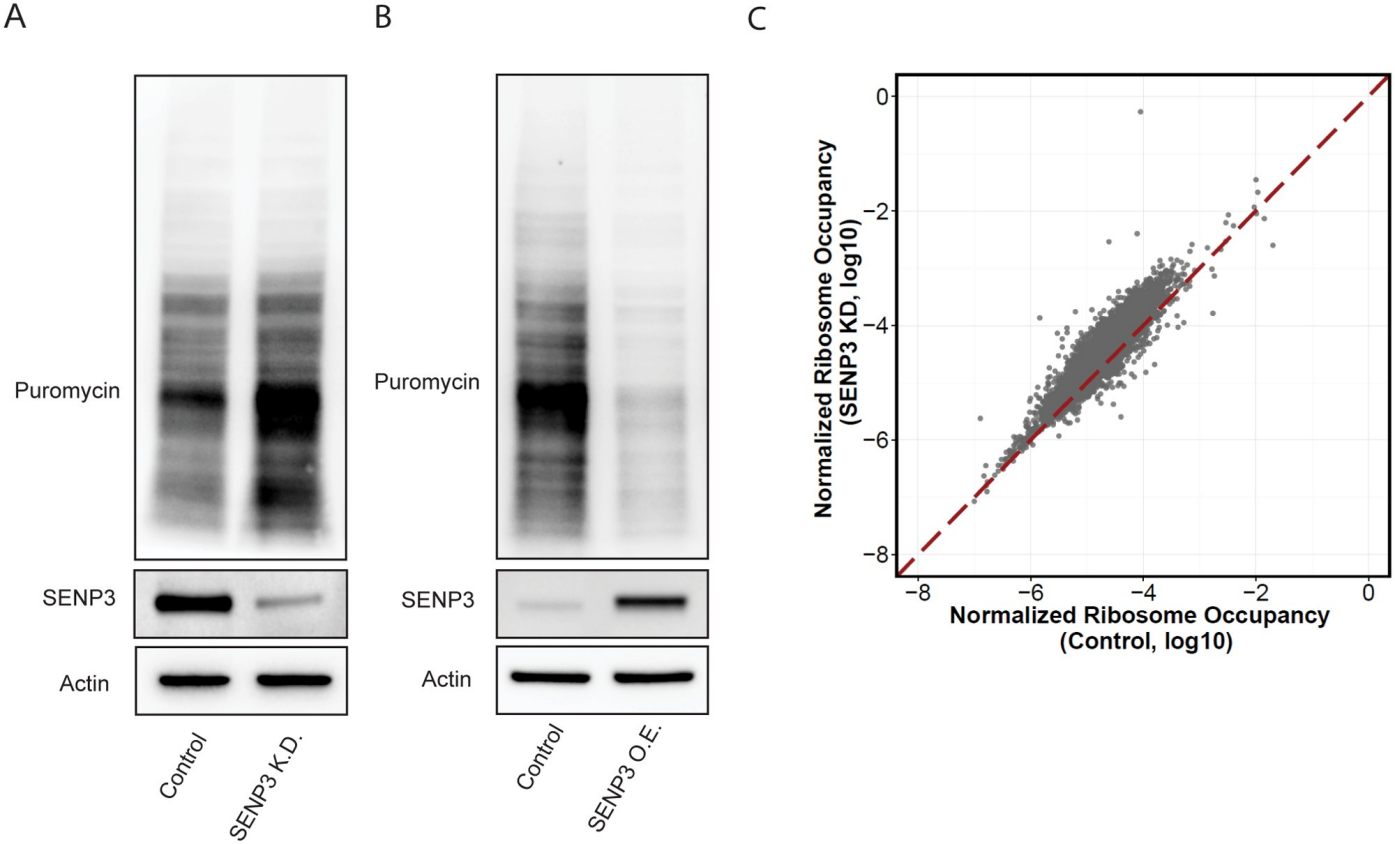
SENP3 silencing restores host protein translation. (A) Immunoblotting of puromycin-labelled newly-synthesized proteins in HepG2.215 control cells and HepG2.215 SENP3 K.D. cells. (B) Immunoblotting of puromycin-labelled newly-synthesized proteins in HepG2.215 control cells and HepG2.215 SENP3 O.E. cells. (C) Normalized ribosome occupancy in HepG2.215 control and HepG2.215 SENP3 K.D. cells.

To measure global translation activity, we labeled the newly synthesized proteins with puromycin, an antibiotic that enters the A site of ribosome and causes puromycylation of nascent polypeptide, leading to premature chain release, followed by immunoblotting using anti-puromycin antibody. The translation level in HepG2.215 cells is lower than that of HepG2 cells (S6A Fig). Knockdown of SENP3 enhanced protein translation in HepG2.215 cells but not in HepG2 cells (Fig 3A, S6B Fig). On the other hand, ectopic expression of SENP3 diminished protein translation (Fig 3B). We then performed ribosome protected fragments deep sequencing (Ribo-seq)[22, 35]. Analysis of the Ribo-seq data showed that ribosome occupancy of host genome is higher in SENP3 knockdown cells, suggesting that SENP3 knockdown increased the rate of host protein translation in HBV positive cells (Fig 3C, S7 Fig).

### IQGAP2 is a de-SUMOylation target of SENP3

**Fig 4:**
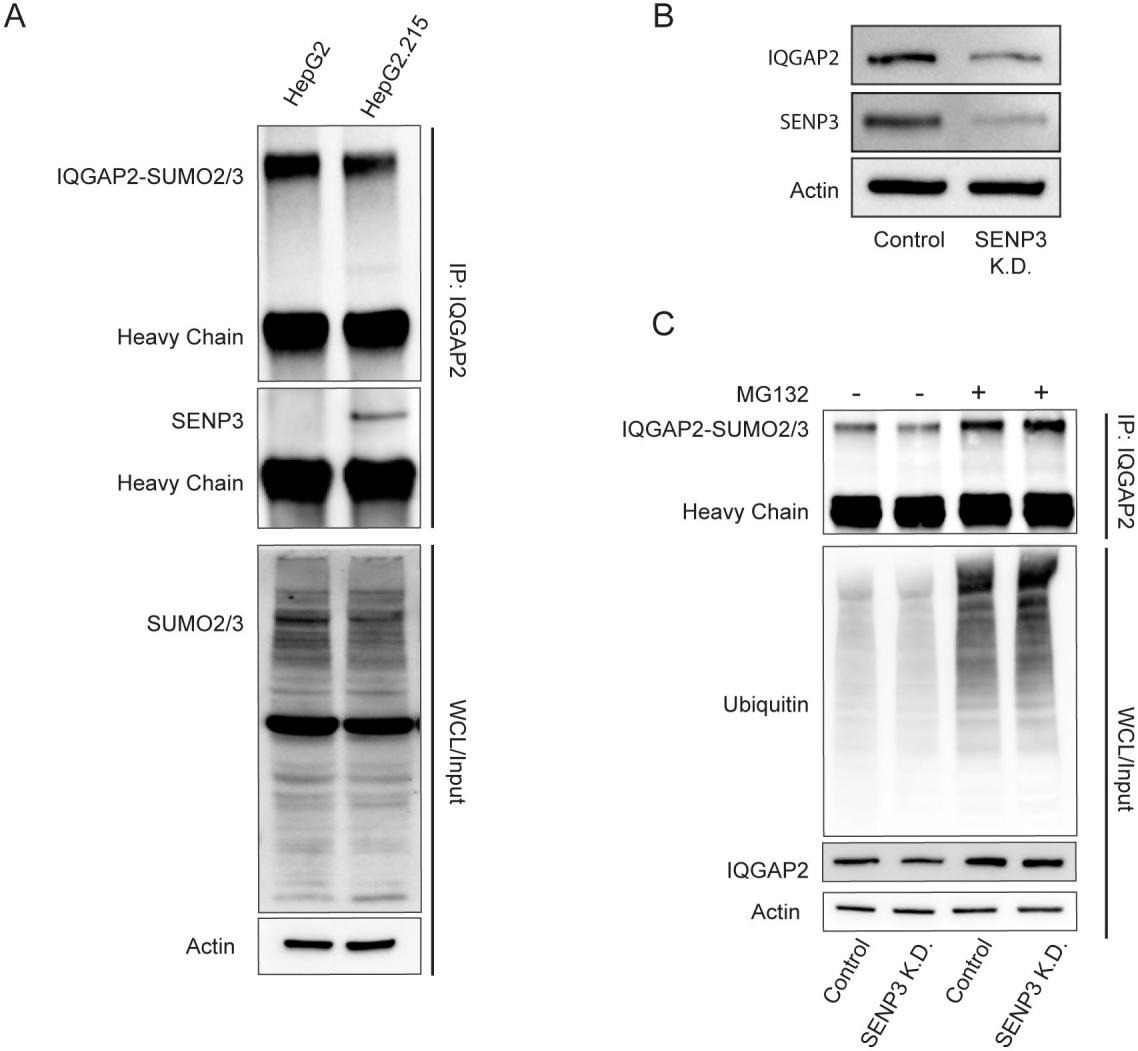
IQGAP2 is a de-SUMOylation target of SENP3. (A) Upper: immunoprecipitation (IP) with an anti-IQGAP2 antibody in HepG2 and HepG2.215 cells followed by immunoblotting of SUMO2/3 and SENP3; lower: immunoblotting of SUMO-2/3 in corresponding whole cell lysates (WCL). (B) Immunoblotting of IQGAP2 in HepG2.215 control and HepG2.215 SENP3 K.D. cells. (C) Upper: immunoprecipitation (IP) with an anti-IQGAP2 antibody followed by immunoblotting of SUMO-2/3 in HepG2.215 control and HepG2.215 SENP3 K.D. cells without MG132 treatment (1^st^ and 2^nd^ lanes), HepG2.215 control and HepG2.215 SENP3 K.D. cells treated with 1 μM MG132 for 6 hrs (3^rd^ and 4^th^ lanes); lower: immunoblotting of ubiquitin and IQGAP2 in corresponding whole cell lysates (WCL).

To identify potential de-SUMOylating target of SENP3 in HBV infected cells, we performed immunoprecipitation (IP) with anti-SENP3 antibody followed by protein mass spectrometry, which identified IQ motif containing GTPase activating protein 2 (IQGAP2) as having the highest affinity with SENP3 (Table 1). IP with anti-IQGAP2 antibody validated that SENP3 is bound to IQGAP2 in HepG2.215 cells but not in HepG2 cells (Fig 4A, upper panel). Immunoblotting with anti-SUMO2/3 antibody on IQGAP2 IP samples confirmed that IQGAP2 could be modified by SUMO2/3 (Fig 4A, upper panel). The IQGAP2 level in HepG2.215 cells was reduced by SENP3 knockdown (Fig 4B). Degradation of SUMOylated IQGAP2 were rescued by MG132 treatment that stalls proteasome activity (Fig 4C). Hence, IQGAP2 is a target of SENP3, and downregulation of SENP3 in HBV-infected cells facilitates IQGAP2 degradation.

**Table 1:** Top 10 SENP3 binding proteins identified from HepG2.215 cells using protein mass spectrometry following immunoprecipitation.

Rank	Protein	Description	Number of unique peptides to the protein
1	IQGAP2_HUMAN	IQGAP2_HUMAN IQ motif containing GTPase-activating-like protein IQGAP2; OS = Homo sapiens GN = IQGAP2 PE = 1 SV = 4	16
2	SYEP_HUMAN	Bifunctional glutamate/proline—tRNA ligase; OS = Homo sapiens GN = EPRS PE = 1 SV = 5	16
3	MYH9_HUMAN	Myosin-9; OS = Homo sapiens GN = MYH9 PE = 1 SV = 4	13
4	RRBP1_HUMAN	Ribosome-binding protein 1; OS = Homo sapiens GN = RRBP1 PE = 1 SV = 4	11
5	FAS_HUMAN	Fatty acid synthase; OS = Homo sapiens GN = FASN PE = 1 SV = 3	10
6	DESP_HUMAN	Desmoplakin; OS = Homo sapiens GN = DSP PE = 1 SV = 3	10
7	NU205_HUMAN	Nuclear pore complex protein; OS = Homo sapiens GN = NUP205 PE = 1 SV = 3	9
8	CO3_HUMAN	Complement C3 protein; OS = Homo sapiens GN = C3 PE = 1 SV = 2	9
9	PYR1_HUMAN	CAD protein; OS = Homo sapiens GN = CAD PE = 1 SV = 3	8
10	DSG1_HUMAN	Desmoglein-1; OS = Homo sapiens GN = CAD PE = 1 SV = 2	6

### IQGAP2 silencing reduces viral gene expression and restores host translation

**Fig 5:**
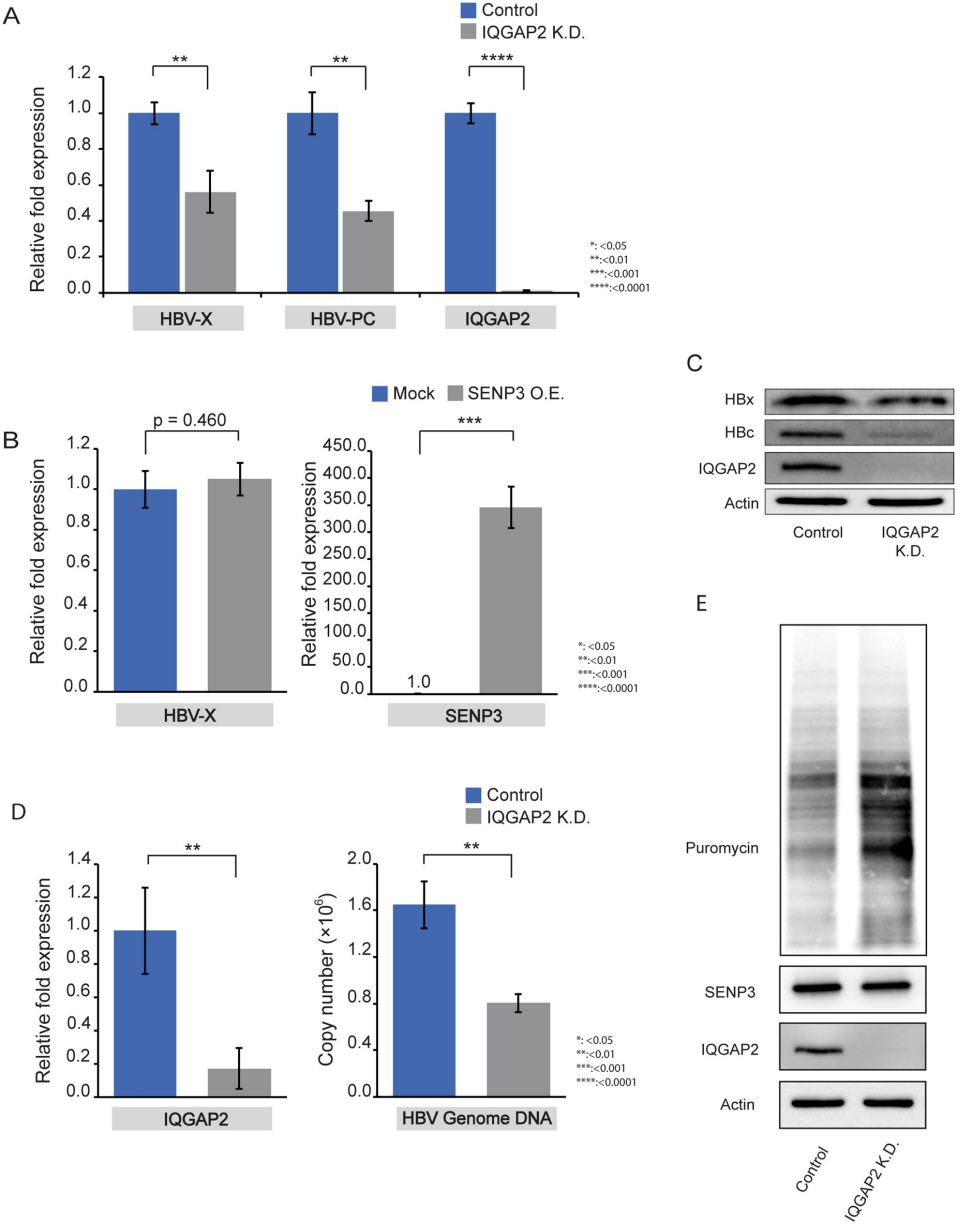
IQGAP2 silencing reduces viral gene expression and restores host translation. (A) RT-qPCR measurements of HBV transcripts amplified by primers HBV-PC and HBV-X in HepG2.215 control cells and HepG2.215 IQGAP2 K.D. cells. Beta-actin was used as internal control; the data were expressed as mean±SD (n = 3). The statistical significance was assessed by Students’ unpaired *t*-test. (B) RT-qPCR measurement of HBV transcripts amplified by primer pair HBV-X in HepG2.215 IQGAP2 K.D. cells with transient transfection of pcDNA3 plasmid (Mock) and RGS-SENP3 plasmid (SENP3 O.E.). Beta-actin was used as internal control; the data were expressed as mean±SD (n = 3). The statistical significance was assessed by Students’ unpaired *t*-test. (C) Immunoblotting of HBx and HBc in HepG2.215 control and HepG2.215 IQGAP2 K.D. cells. (D) Left: RT-qPCR confirmation of IQGAP2 knockdown in HepG2-NTCP cells. Right: qPCR measurements of HBV genome copy numbers in supernatants of HepG2-NTCP cells (control and IQGAP2 K.D.) on Day 7 after HBV infection. Beta-actin (left) and HBV 1.3-mer WT replicon (Addgene Plasmid #65459) (right) were used as internal control. The data were mean±SD (n = 3) and the statistical significance was assessed by Students’ unpaired *t*-test. (E) Immunoblotting of puromycin-labelled proteins in HepG2.215-control and HepG2.215-IQGAP2 K.D. cells.

We found that IQGAP2 knockdown also reduced viral transcription in HepG2.215 and HepAD38 cells (Fig 5A, S8 Fig) shown by RT-qPCR using the same sets of primers. HBV transcription cannot be restored by ectopic expression of wild-type SENP3 in IQGAP2 knockdown cells (Fig 5B), indicating that the effect of SENP3 on HBV gene expression is mediated through IQGAP2. IQGAP2 silencing also decreased HBx and HBc protein levels in HepG2.215 cells (Fig 5C) and reduced HBV genome copy numbers in the supernatants of HepG2-NTCP cells infected with HBV (Fig 5D). Meanwhile, it enhanced host protein translation according to puromycin labeling (Fig 5E).

### The SENP3-IQGAP2 axis mediates Akt phosphorylation in HBV-infected cells

**Fig 6:**
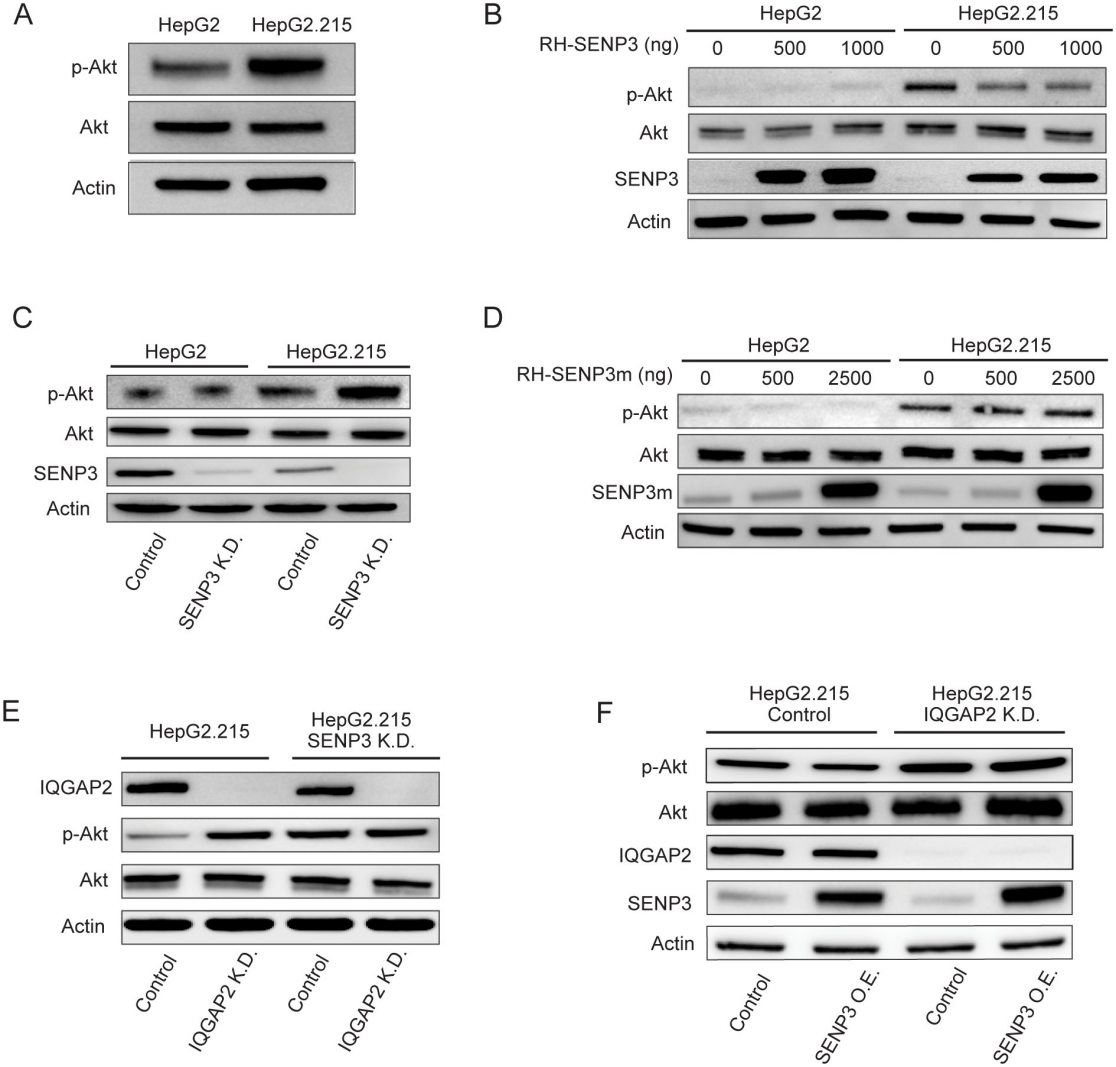
The SENP3-IQGAP2 axis mediates Akt phosphorylation in HBV infected cells. (A-F) Comparison of AKT phosphorylation by immunoblotting in HepG2 and HepG2.215 cells (A), in HepG2 and HepG2.215 cells both with ectopic expression of SENP3 by transient transfection of differing amounts of RH-tagged SENP3 constructs (B), in HepG2-SENP3 K.D. and HepG2.215-SENP3 K.D. cells compared with corresponding control cells (C), in HepG2 and HepG2.215 cells with ectopic expression of SENP3 mutant (C532A) (SENP3m) by transient transfection of differing amounts of RH-tagged SENP3m constructs (D), in HepG2.215 cells with IQGAP2 K.D., SENP3 K.D., and knockdown of both proteins (E), in HepG2.215-control and HepG2.215-IQGAP2 K.D. with and without ectopic expression of SENP3 by transient transfection of RH-tagged SENP3 constructs (F).

Akt phosphorylation is a key mediator linking the phosphatidylinositol 3-kinase (PI3K)/Akt signaling pathway to the translational machinery[36, 37]. Akt activation and PI3K/Akt signaling have also been shown to reduce HBV transcription and replication via hepatocyte nuclear factor 4α (HNF4α)[38, 39]. Therefore, we tested whether the observed effect of decreased SENP3 and IQGAP2 on host translation and HBV gene expression is dependent on Akt activation. Akt phosphorylation level is higher in HepG2.215 cells than in HepG2 cells (Fig 6A). Ectopic expression of SENP3 suppressed Akt phosphorylation in HepG2.215 cells, but not in HepG2 cells, which had relatively low Akt phosphorylation level (Fig 6B). Knockdown of SENP3 elevated Akt phosphorylation in HepG2.215 cells, while no such effect was observed in HepG2 cells (Fig 6C). These results indicate that SENP3 suppresses Akt phosphorylation in HBV-infected cells. On the other hand, blockage of PI3K/Akt/mTOR signaling pathway by treatment with either Rapamycin (mTOR inhibitor) or LY294002 (PI3K inhibitor) had no effect on SENP3 expression level, confirming that SENP3 is upstream of Akt phosphorylation (S9 Fig). Ectopic expression of SENP3 mutant (C532A) had little effect on Akt phosphorylation in either HepG2 or HepG2.215 cells (Fig 6D), confirming that the suppression of Akt phosphorylation by SENP3 is dependent on its de-SUMOylation activity. Like SENP3 knockdown, IQGAP2 knockdown also increased Akt phosphorylation in HepG2.215 cells (Fig 6E). Knockdown or ectopic expression of SENP3 in HepG2.215 cells no longer impacted Akt phosphorylation when IQGAP2 was silenced (Fig 6E and 6F), suggesting that SENP3 regulation of Akt phosphorylation is dependent on IQGAP2. Hence, our data suggested that the SENP3-IQGAP2 SUMOylation axis mediates Akt phosphorylation upon HBV infection.

### PI3K/Akt pathway promote host translation and suppresses HBV gene transcription

**Fig 7:**
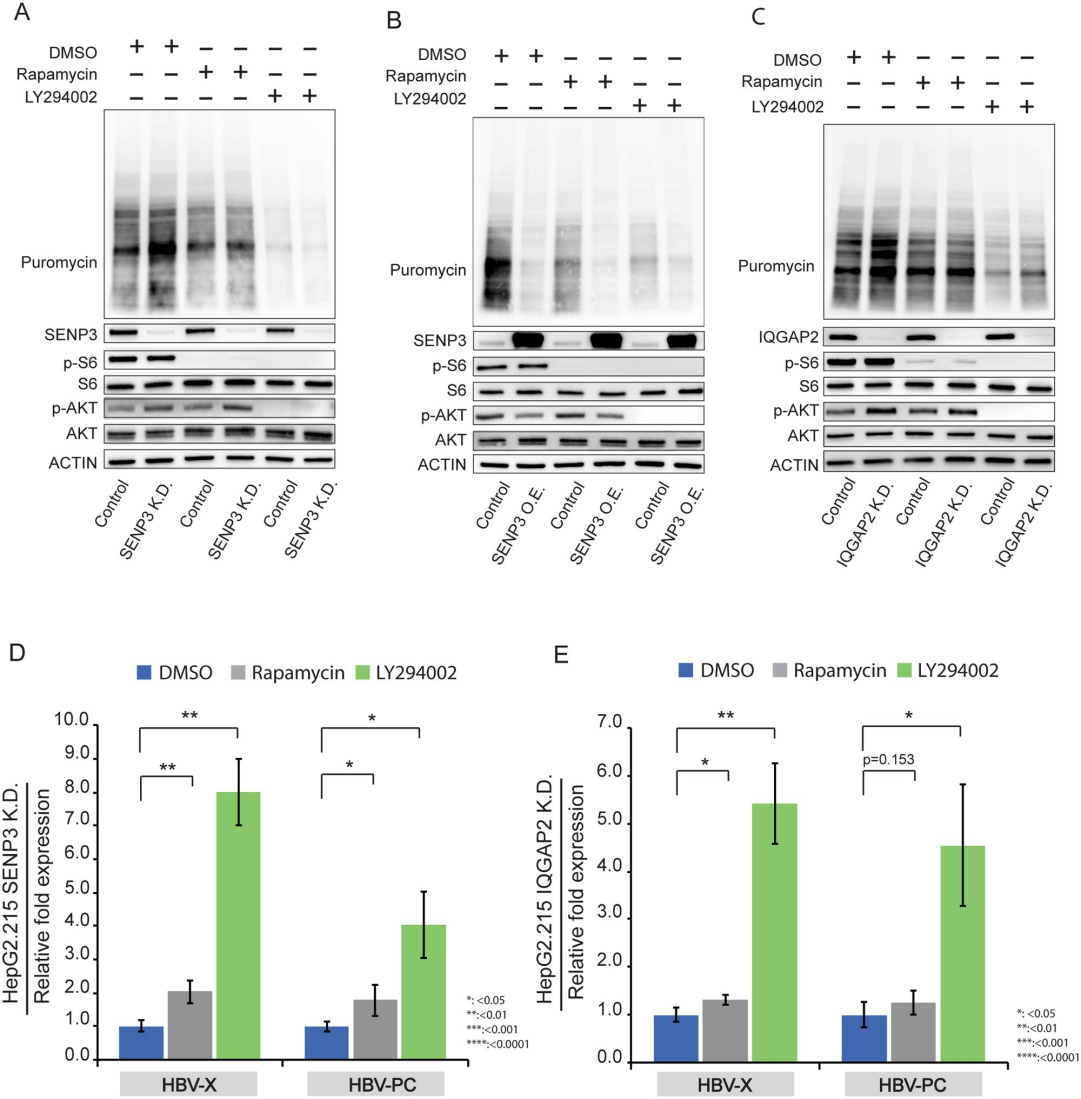
PI3K/Akt pathway promote host translation and suppresses HBV gene transcription. (A) Immunoblotting of puromycin-labelled proteins in HepG2.215 control and HepG2.215-SENP3 K.D. cells after being treated for 6 hrs with DMSO (as control), Rapamycin (20 nM) and LY294002 (20 μM). Phosphorylated S6 (P-S6) and phosphorylated Akt (p-AKT) were used to indicate inhibition of mTOR and PI3K respectively. (B) Immunoblotting of puromycin-labelled proteins in HepG2.215 control and HepG2.215 SENP3 O.E. cells after being treated for 6 hrs with DMSO (as control), Rapamycin (20 nM) and LY294002 (20 μM). Phosphorylated S6 (P-S6) and phosphorylated Akt (p-AKT) were used to indicate inhibition of mTOR and PI3K respectively. (C) Immunoblotting of puromycin-labelled proteins in HepG2.215 control and HepG2.215 IQGAP2 K.D. cells after being treated for 6 hrs with DMSO (as control), Rapamycin (20 nM) and LY294002 (20 μM). Phosphorylated S6 (P-S6) and phosphorylated Akt (p-AKT) were used to indicate inhibition of mTOR and PI3K respectively. (D) RT-qPCR measurements of HBV transcripts amplified by primers HBV-PC and HBV-X in HepG2.215 SENP3 K.D. cells after being treated for 6 hrs with DMSO (as control), Rapamycin (20 nM) and LY294002 (20 μM). Beta-actin was used as internal control. Data were mean±SD (n = 3) and the statistical significance was assessed by Students’ unpaired *t*-test. (E) RT-qPCR measurements of HBV transcripts amplified by primers HBV-PC and HBV-X in HepG2.215 IQGAP2 K.D. cells after being treated for 6 hrs with DMSO (as control), Rapamycin (20 nM) and LY294002 (20 μM). Beta-actin was used as internal control. Data were mean±SD (n = 3) and the statistical significance was assessed by Students’ unpaired *t*-test.

The enhancement of host protein synthesis by SENP3 or IQGAP2 knockdown in HepG2.215 cells was partially abrogated by Rapamycin treatment, which inhibits mTOR phosphorylation (Fig 7A–7C), and was almost completely abrogated by LY294002 treatment, which is an inhibitor further upstream of the PI3K/Akt pathway (Fig 7A–7C). Therefore, the effect of SENP3 and IQGAP2 on host translation in HBV-infected cells is dependent on Akt activity.

We had shown that SENP3 (Fig 2A) or IQGAP2 knockdown (Fig 5A) suppresses HBV transcription in HepG2.215 cells. Inhibition of the PI3K/Akt pathway by Rapamycin or LY294002 treatment rescued HBV gene transcription in HepG2.215 cells with either SENP3 or IQGAP2 knockdown (Fig 7D and 7E). The effect of LY294002 is stronger than Rapamycin, consistent with the fact that LY294002 is further upstream and more effective at suppressing host translation, hence activating HBV expression.

## Discussion

**Fig 8:**
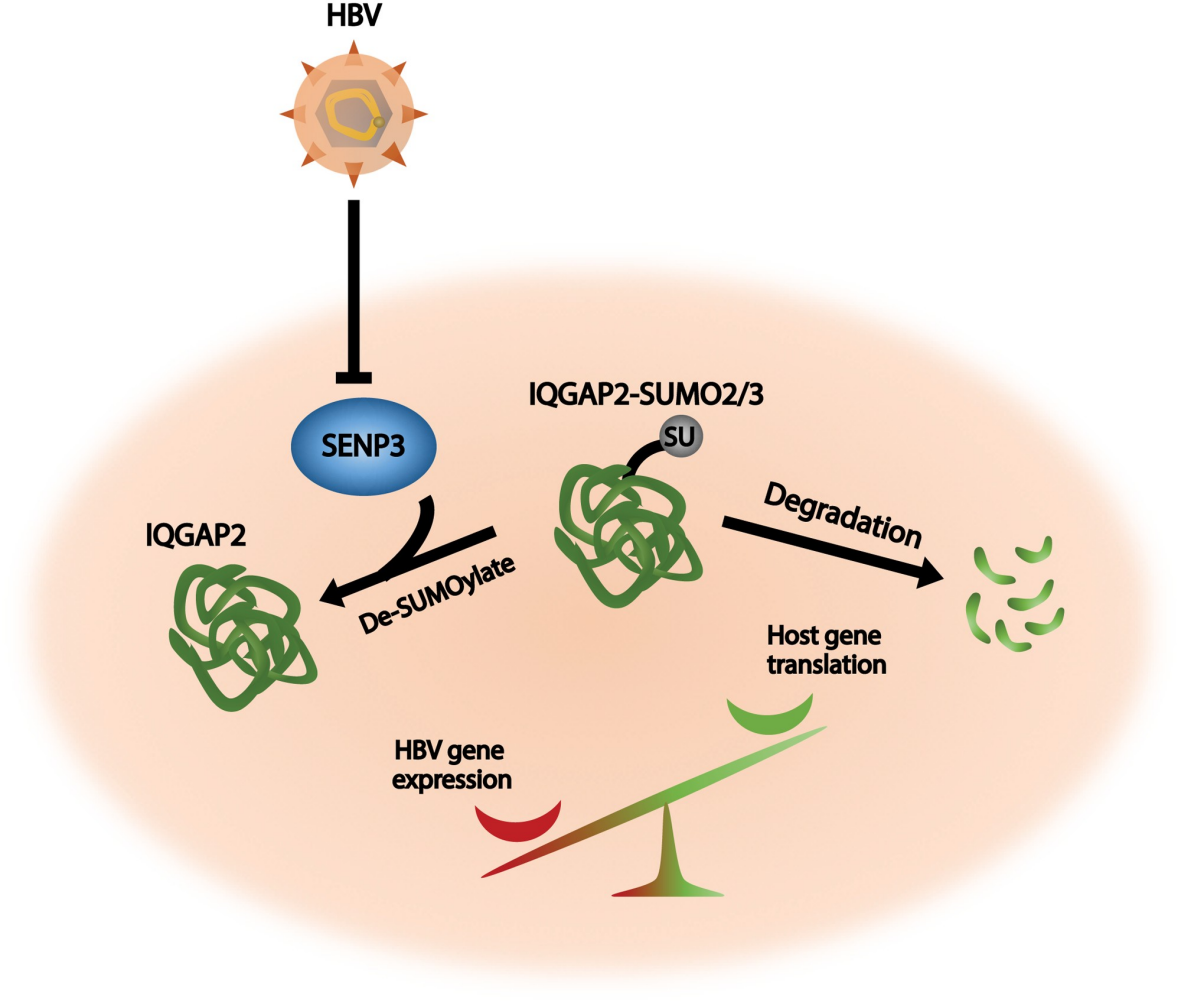
Graphical summary. Upon hepatitis B virus (HBV) infection, hepatocytes downregulate SENP3 to promote SUMOylation and subsequent degradation of IQGAP2. The SENP3-IQGAP2 de-SUMOylation axis is a host defense mechanism that restores host protein translation and suppresses HBV gene expression.

HBV exploits host resources via a complex network of virus-host interactions to satisfy its replication needs and persist in the hepatocytes[40]. For example, HBV regulatory protein HBx promotes HBV genome transcription by hijacking DDB1- containing E3 ligase to compete with host chromosome[7]. In response, the PI3K/Akt signaling in the host cells can be activated by HBV infection, which reduces HBV replication and promotes host survival via factors such as hepatocyte nuclear factor 4α (HNF4α)[38, 39]. However, the exact mechanism of Akt activation in HBV-infected host has been unclear. Here, we demonstrate that hepatocytes respond to HBV infection by downregulating SUMO-2/3-specific peptidase SENP3, which subsequently leads to SUMOylation and degradation of IQGAP2, and upregulation of Akt phosphorylation. This SUMOylation-mediated signaling axis suppresses HBV gene expression and promotes host translation (Fig 8), hence aiding host cell survival and making HBV largely non-cytopathic.

SENP3 is a member of SENP family which deconjugates SUMO2/3-modified proteins and functions as a sensor to cellular stress to maintain normal physiology[41]. An increase in the SENP3 level has been associated with different stress conditions and diseases. For example, SENP3 deSUMOylates the HIF1α coactivator P300 to increase angiogenesis under hypoxia [42], deSUMOylates promyelocytic leukemia in response to mild oxidative stress[18], and de-SUMOylating FOXC2 to facilitate epithelial-mesenchymal transition in gastric cancer cells [17]. The role of SENP3 in stress response is likely context-dependent. It has been shown that many viruses, including HBV, can induce oxidative stress in the host cells[43, 44]. Thus, the exact mechanism that lead to SENP3 downregulation during HBV infection is likely to be stress-related.

Very few SENP3 substrates have been identified so far. IQGAP2 is a scaffolding protein containing three IQ motifs and GTPase activating protein (GAP) related domains[45, 46]. It is primarily expressed in the liver, regulating cellular morphology and motility via interaction with cytoskeleton, cell adhesion molecules *etc*[46, 47]. Loss of IQGAP2 has been associated with liver, gastric and prostate cancers[48–50]. It has been reported that IQGAP2 inhibits Akt activation in DU145 cells, which upregulates E-cadherin[48], consistent with our observation that suppression of IQGAP2 enhances PI3K/Akt signaling. However, the exact mechanism of how IQGAP2 regulates Akt phosphorylation remains to be elucidated. IQGAP2 is also involved in interferon antiviral response to Hepatitis C virus (HCV) via the NF-κB pathway[51]. Thus, its protective role against viral infection may depend on the virus type.

Many viruses hijack host translational machinery for replication and eventually reach a balance[52, 53]. Our ribo-seq data indicate the ribosome occupancy of host genes increased after downregulation of SENP3. It is possible that restoration of host translation activity can negatively impact the translation of viral mRNA in addition to transcription inhibition by Akt signaling. However, the ribo-seq data did not have enough sequencing depth to robustly evaluate viral mRNA translation, which may be due to relatively low viral mRNA levels caused by Akt suppression[38].

The SENP3-IQGAP2 SUMOylation axis is probably not the only defensive mechanism that host cells can modulate against viral particles. It is also possible that SENP3 may be a central viral stress sensor and protector of host translation—it responds to different viral infections by degrading IQGAP2 to boost Akt phosphorylation. It will be interesting to test whether SENP3 also responds to other viral species and carry out a more comprehensive investigation into anti-viral translational reprogramming. A better understanding of the SENP3-IQGAP2 SUMOylation axis, including how HBV infection downregulates SENP3 and how IQGP2 regulates Akt signaling, may lead to novel therapeutic strategies against HBV with the benefit of restoring normal liver functions.

## Supporting information

S1 Table Detailed information for selected reagents. (A) MISSION shRNA constructs (Sigma-Aldrich) used for gene knockdown. (B) Primary antibodies used for immunoblotting and immonofluorescence staining.
(PDF)

S2 Table Sequences of primers for amplifying various combinations of HBV transcripts. (PDF)

S1 Fig Representative immunofluorescence staining image of HBV-infected HepG2-NTCP cells. Scale bar indicates 100 μm.
(PDF)

S2 Fig H&E staining of normal liver section and HBV-infected liver section. Normal liver tissue was provided by the Liver Lesion Database at the Duke. The tissue was from the normal liver adjacent to a focus of metastatic colon cancer, and was > 1 cm from the tumor mass. The HBV-infected liver tissue was provided by Duke Translational Research Institute Biobank (BRPC-15-876). The lab tests showed that alanine aminotransferase (ALT) level was 53 U/L, and bilirubin level 1.1 mg/dL. Scale bar indicates 200 μm.
(PDF)

S3 Fig SENP3 expression in HepG2 and HepG2.215 cells. (A) RT-qPCR measurement of SENP3 mRNA in HepG2 and HepG2.215 cells. Beta-actin was used as internal control. Beta-actin was used as internal control. Data were mean±SD (n = 3) and the statistical significance was assessed by Students’ unpaired t-test. (B) Immunoblotting of SENP3 in HepG2 and HepG2.215 cells. Before RNA or protein extraction, both cells are cultured under the exact same condition and incubated for the exact same durations after being seeded.
(PDF)

S4 Fig SENP3 expression in HepG2 cells inducibly expressing HBx (HepG2-HBx cells). (A) RT-qPCR measurement of mRNA levels of SENP3 and HBx in HepG2-HBx cells with and without treatment with doxycycline (500 ng/ml) for 5 days. Primer pair HBV-X was used to amplify the X mRNAs in the cells to indicate the success of doxycycline induction. Beta-actin was used as internal control. Data were mean±SD from two biological repeats and the statistical significance was assessed by Students’ unpaired t-test. (B) Immunoblotting of SENP3 in HepG2-HBx cells with and without doxycycline induction.
(PDF)

S5 Fig SENP3 silencing suppresses HBV replication. (A) RT-qPCR measurements of HBV transcripts amplified by primers HBV-X and HBV-PC in HepAD38-control cells and HepAD38-SENP3 K.D. cells. Beta-actin was used as internal control; the data were expressed as mean±SD (n = 3). Statistical significance was assessed by Students’ unpaired t-test. (B) Left: RT-qPCR measurement of HBV transcripts amplified by primer HBV-PC after transcient transcription of RGS-SENP3 plasmid and RGS-SENP3m plasmid for ectopic expression of SENP3 or SENP3 mutant (SENP3m) in HepG2.215 cells. Right: RT-qPCR measurement of SENP3 or SENP3m in HepG2.215 cells to indicate the success of ectopic expression. Beta-actin was used as internal control. Beta-actin was used as internal control; the data were expressed as mean±SD (n = 3). Statistical significance was assessed by Students’ unpaired t-test. (C) Left: HBsAg levels in supernatants of HepG2-NTCP cells (control and SENP3 K.D.) after HBV infection measured by ELISA. Right: HBeAg levels in supernatants of HepG2-NTCP cells (control and SENP3 K.D.) after HBV infection measured by ELISA. Data were mean±SD (n = 3). Statistical significance was assessed by Students’ unpaired t-test.
(PDF)

S6 Fig Translation levels in HepG2 cells. (A) Immunoblotting of puromycin-labelled proteins in HepG2 and HepG2.215 cells. (B) Immunoblotting of puromycin-labelled proteins in HepG2-control and HepG2-SENP3 K.D. cells.
(PDF)

S7 Fig Ribo-seq quality control. (A) Quality control of Ribo-seq library from HepG2.215-control cells. (B) Quality control of Ribo-seq library from HepG2.215-SENP3 K.D. cells.
(PDF)

S8 Fig IQGAP2 silencing suppresses HBV transcription in HepAD38. RT-qPCR measurements of HBV transcripts amplified by primers HBV-X and HBV-PC in HepAD38 control cells and IQGAP2K.D. cells. Beta-actin was used as internal control; the data were expressed as mean±SD (n = 3). Statistical significance was assessed by Students’ unpaired t-test.
(PDF)

S9 Fig SENP3 level in HepG2 and HepG2.215 cells after treatment with Rapamycin and LY294002. Immunoblotting of SENP3 in HepG2 and HepG2.215 cells after being treated with Rapamycin (20 nM) to inhibit mTOR and LY294002 (20 μM) to inhibit PI3K. Phosphorylated S6 (P-S6) and phosphorylated Akt (p-AKT) were used to indicate inhibition of mTOR and PI3K, respectively.
(PDF)
