# Supplementary material for: SENP3-mediated host defense response contains HBV replication and restores protein synthesis
Source: PLoS One. 2019 Jan 14;14(1):e0209179. doi: 10.1371/journal.pone.0209179 (PMC6331149; doi:10.1371/journal.pone.0209179)
Supplement: S1 Table — (A) MISSION shRNA constructs (Sigma-Aldrich) used for gene knockdown. (B) Primary antibodies used for immunoblotting and immonofluorescence staining. (PDF) [file pone.0209179.s001.pdf]

## S1 Table. Detailed information for selected reagents

**(A)** MISSION shRNA constructs (Sigma-Aldrich) used for gene knockdown

| Target Gene | TRC Number | Product Details |
|---|---|---|
| **SENP3** | TRCN0000004109 | **Region**: CDS **TRC Version**: 1<br>**Sequence**:CCGGCCTCGCTGACATTCCACTGGACTCGAGTCCAGTGG AATGTCAGCGAGGTTTTT |
| **IQGAP2** | TRCN0000414490 | **Region**: CDS **TRC Version**: 2<br>**Sequence**:CCGGACTAGAGGTTGCACGATTAAGCTCGAGCTTAATCGT GCAACCTCTAGTTTTTTG |

**(B)** Primary antibodies used for immunoblotting and immonofluorescence staining

| Target Protein | Antibody | Vendor |
|---|---|---|
| **SENP3** | SENP3 (D20A10) Rabbit mAb | Cell Signaling Technology |
| **Actin** | β-Actin (13E5) Rabbit mAb | Cell Signaling Technology |
| **P-Akt** | Phospho-Akt Ser473 (D9E) XP¨ Rabbit mAb | Cell Signaling Technology |
| **Akt** | Akt (pan) (C67E7) Rabbit mAb | Cell Signaling Technology |
| **IQGAP2** | IQGAP2 (D1X8U) Rabbit mAb | Cell Signaling Technology |
| **Ubiquitin** | Ubiquitin (P4D1) Mouse mAb | Cell Signaling Technology |
| **P-S6** | Phospho-S6 Ribosomal Protein (Ser235/236) (D57.2.2E) Rabbit mAb | Cell Signaling Technology |
| **S6** | S6 Ribosomal Protein (5G10) Rabbit mAb | Cell Signaling Technology |
| **Puromycin** | Anti-puromycin antibody clone 12D10 | Sigma-Aldrich |
| **Sumo 2/3** | Anti-Sumo 2+3 antibody [8A2] | Abcam |
| **HBx** | Anti-Hepatitis B Virus X antigen antibody [3F6-G10] | Abcam |
| **HBc** | Anti-Hepatitis B Virus Core Antigen antibody [14E11] | Abcam |
| **HBsAg** | Anti-Hepatitis B Virus Surface Antigen (Ad/Ay) antibody (ab9193) | Abcam |

**(C)** Primers used for RT-qPCR

| Target Gene | Forward (5' to 3') | Reverse (5' to 3') |
|---|---|---|
| **SENP3** | ACAGAACTGGCTCAATGACC | TCCACCTTTTCACCCCATC |
| **IQGAP2** | CCCTTGATCTACTGCCTTATGG | GGCTGGATTCATGTACCGATAG |
| **HBV-PC** | GGTCTGCGCACCAGCACC | GAACTTTAGGCCCATATTAGTG |
| **HBV-X** | CGTCTGTGCCTTCTCATCTG | ACATTGCTGAGAGTCCAAGAG |
| **Beta-actin** | CGCGAGAAGATGACCCAGAT | ACAGCCTGGATAGCAACGTACAT |
