# Supplementary material for: SENP3-mediated host defense response contains HBV replication and restores protein synthesis
Source: PLoS One. 2019 Jan 14;14(1):e0209179. doi: 10.1371/journal.pone.0209179 (PMC6331149; doi:10.1371/journal.pone.0209179)
Supplement: S2 Table — (PDF) [file pone.0209179.s002.pdf]

S2 Table. Sequences of primers used to amplify various combinations of HBV transcripts

GenBank accession number of the reference HBV genome: **KX827302.1**

Primer	Sequence (5' to 3')	Mapping region (nt)	Amplified transcripts
HBV-PC	F: GGTCTGCGCACCAGCACC<br>R: GAACTTTAGGCCCATATTAGTG	1796-2191	Precore mRNA
HBV-X	F: CGTCTGTGCCTTCTCATCTG<br>R: ACATTGCTGAGAGTCCAAGAG	1550-1683	Precore mRNA; pregenomic mRNA; 2.1-kb mRNA; 2.4-kb mRNA; X mRNA
