# Supplementary material for: SENP3-mediated host defense response contains HBV replication and restores protein synthesis
Source: PLoS One. 2019 Jan 14;14(1):e0209179. doi: 10.1371/journal.pone.0209179 (PMC6331149; doi:10.1371/journal.pone.0209179)
Supplement: S1 Fig — Scale bar indicates 100 μm. (PDF) [file pone.0209179.s003.pdf]

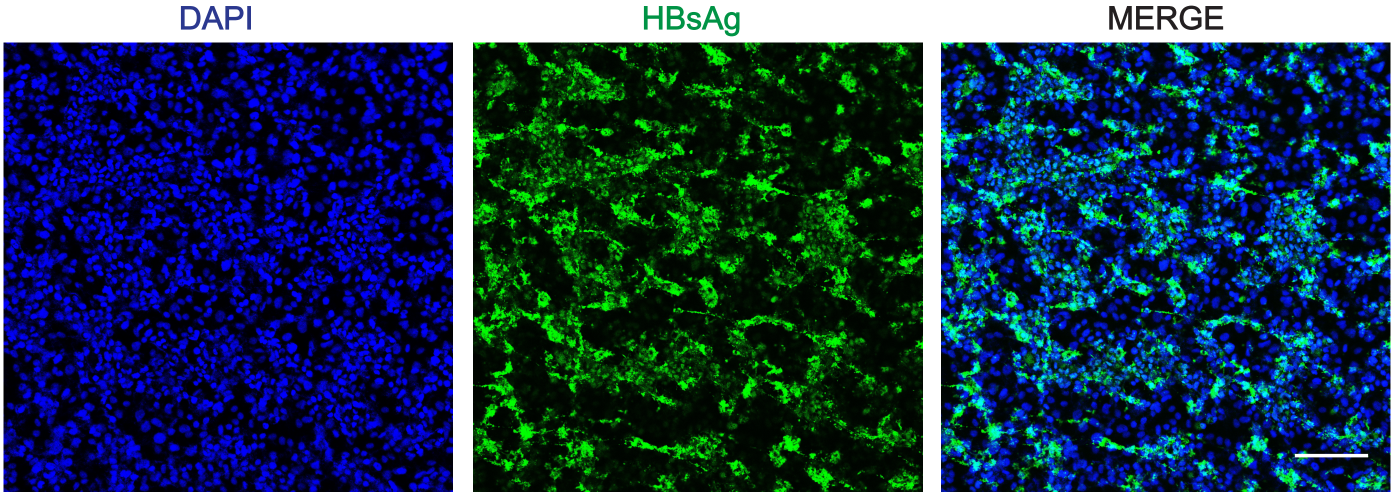

S1 Fig. Representative immunofluorescence staining image of HBV-infected HepG2-NTCP cells. Scale bar indicates 100 µm.
