# Supplementary material for: SENP3-mediated host defense response contains HBV replication and restores protein synthesis
Source: PLoS One. 2019 Jan 14;14(1):e0209179. doi: 10.1371/journal.pone.0209179 (PMC6331149; doi:10.1371/journal.pone.0209179)
Supplement: S2 Fig — Normal liver tissue was provided by the Liver Lesion Database at the Duke. The tissue was from the normal liver adjacent to a focus of metastatic colon cancer, and was > 1 cm from the tumor mass. The HBV-infected liver tissue was provided by Duke Translational Research Institute Biobank (BRPC-15-876). The lab tests showed that alanine aminotransferase (ALT) level was 53 U/L, and bilirubin level 1.1 mg/dL. Scale bar indicates 200 μm. (PDF) [file pone.0209179.s004.pdf]

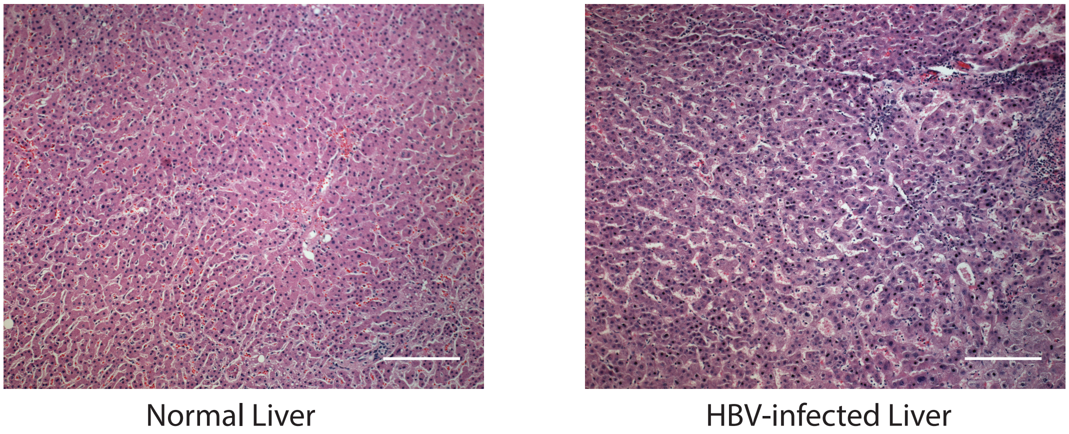

S2 Fig. H&E staining of normal liver section and HBV-infected liver section.
Normal liver tissue was provided by the Liver Lesion Database at the Duke. The tissue was from the normal liver adjacent to a focus of metastatic colon cancer, and was > 1 cm from the tumor mass. The HBV-infected liver tissue was provided by Duke Translational Research Institute Biobank (BRPC-15-876). The lab tests showed that alanine aminotransferase (ALT) level was 53 U/L, and bilirubin level 1.1 mg/dL. Scale bar indicates 200 µm.
