# Supplementary material for: SENP3-mediated host defense response contains HBV replication and restores protein synthesis
Source: PLoS One. 2019 Jan 14;14(1):e0209179. doi: 10.1371/journal.pone.0209179 (PMC6331149; doi:10.1371/journal.pone.0209179)
Supplement: S3 Fig — (A) RT-qPCR measurement of SENP3 mRNA in HepG2 and HepG2.215 cells. Beta-actin was used as internal control. Beta-actin was used as internal control. Data were mean±SD (n = 3) and the statistical significance was assessed by Students’ unpaired t-test. (B) Immunoblotting of SENP3 in HepG2 and HepG2.215 cells. Before RNA or protein extraction, both cells are cultured under the exact same condition and incubated for the exact same durations after being seeded. (PDF) [file pone.0209179.s005.pdf]

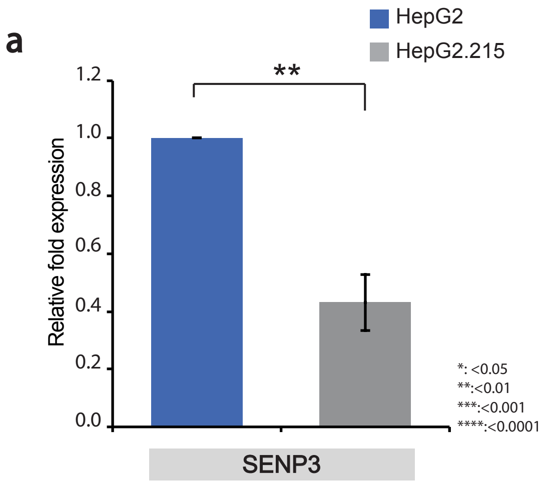

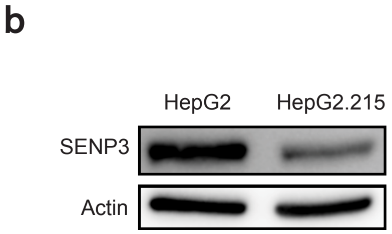

S3 Fig. SENP3 expression in HepG2 and HepG2.215 cells.
(A) RT-qPCR measurement of SENP3 mRNA in HepG2 and HepG2.215 cells. Beta-actin was used as internal control. Beta-actin was used as internal control. Data were mean±SD (n=3) and the statistical significance was assessed by Students' unpaired t-test. (B) Immunoblotting of SENP3 in HepG2 and HepG2.215 cells. Before RNA or protein extraction, both cells are cultured under the exact same condition and incubated for the exact same durations after being seeded.
