# Supplementary material for: SENP3-mediated host defense response contains HBV replication and restores protein synthesis
Source: PLoS One. 2019 Jan 14;14(1):e0209179. doi: 10.1371/journal.pone.0209179 (PMC6331149; doi:10.1371/journal.pone.0209179)
Supplement: S4 Fig — (A) RT-qPCR measurement of mRNA levels of SENP3 and HBx in HepG2-HBx cells with and without treatment with doxycycline (500 ng/ml) for 5 days. Primer pair HBV-X was used to amplify the X mRNAs in the cells to indicate the success of doxycycline induction. Beta-actin was used as internal control. Data were mean±SD from two biological repeats and the statistical significance was assessed by Students’ unpaired t-test. (B) Immunoblotting of SENP3 in HepG2-HBx cells with and without doxycycline induction. (PDF) [file pone.0209179.s006.pdf]

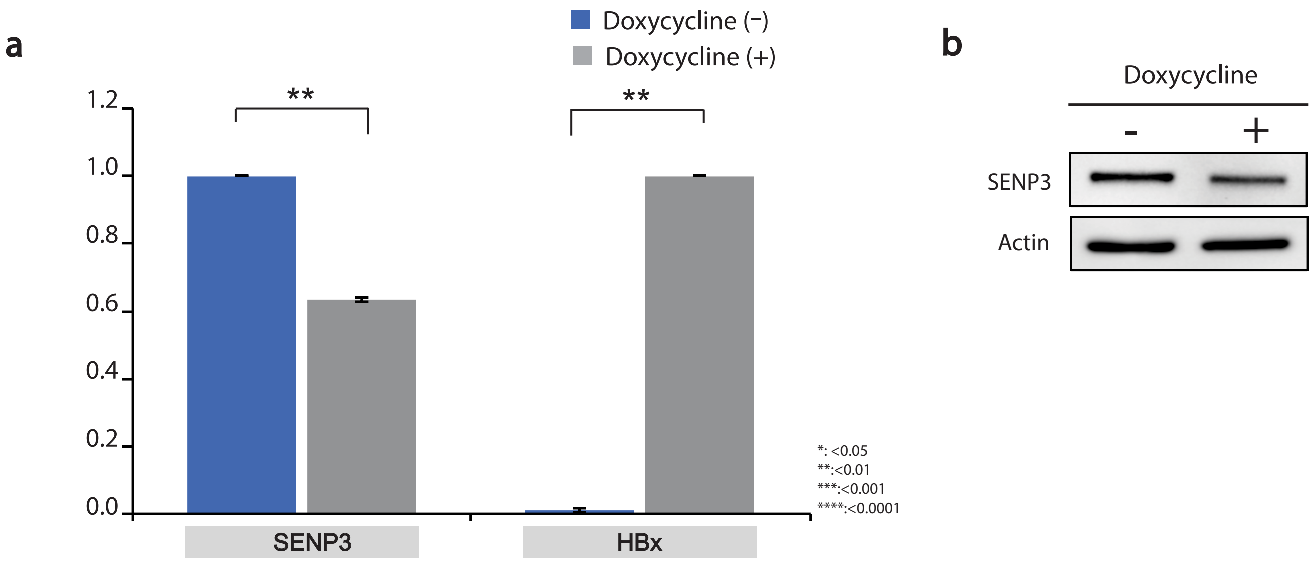

S4 Fig. SENP3 expression in HepG2 cells inducibly expressing HBx (HepG2-HBx cells).
(A) RT-qPCR measurement of mRNA levels of SENP3 and HBx in HepG2-HBx cells with and without treatment with doxycycline (500 ng/ml) for 5 days. Primer pair HBV-X was used to amplify the X mRNAs in the cells to indicate the success of doxycycline induction. Beta-actin was used as internal control. Data were mean±SD from two biological repeats and the statistical significance was assessed by Students' unpaired t-test. (B) Immunoblotting of SENP3 in HepG2-HBx cells with and without doxycycline induction.
