# Supplementary material for: SENP3-mediated host defense response contains HBV replication and restores protein synthesis
Source: PLoS One. 2019 Jan 14;14(1):e0209179. doi: 10.1371/journal.pone.0209179 (PMC6331149; doi:10.1371/journal.pone.0209179)
Supplement: S5 Fig — (A) RT-qPCR measurements of HBV transcripts amplified by primers HBV-X and HBV-PC in HepAD38-control cells and HepAD38-SENP3 K.D. cells. Beta-actin was used as internal control; the data were expressed as mean±SD (n = 3). Statistical significance was assessed by Students’ unpaired t-test. (B) Left: RT-qPCR measurement of HBV transcripts amplified by primer HBV-PC after transcient transcription of RGS-SENP3 plasmid and RGS-SENP3m plasmid for ectopic expression of SENP3 or SENP3 mutant (SENP3m) in HepG2.215 cells. Right: RT-qPCR measurement of SENP3 or SENP3m in HepG2.215 cells to indicate the success of ectopic expression. Beta-actin was used as internal control. Beta-actin was used as internal control; the data were expressed as mean±SD (n = 3). Statistical significance was assessed by Students’ unpaired t-test. (C) Left: HBsAg levels in supernatants of HepG2-NTCP cells (control and SENP3 K.D.) after HBV infection measured by ELISA. Right: HBeAg levels in supernatants of HepG2-NTCP cells (control and SENP3 K.D.) after HBV infection measured by ELISA. Data were mean±SD (n = 3). Statistical significance was assessed by Students’ unpaired t-test. (PDF) [file pone.0209179.s007.pdf]

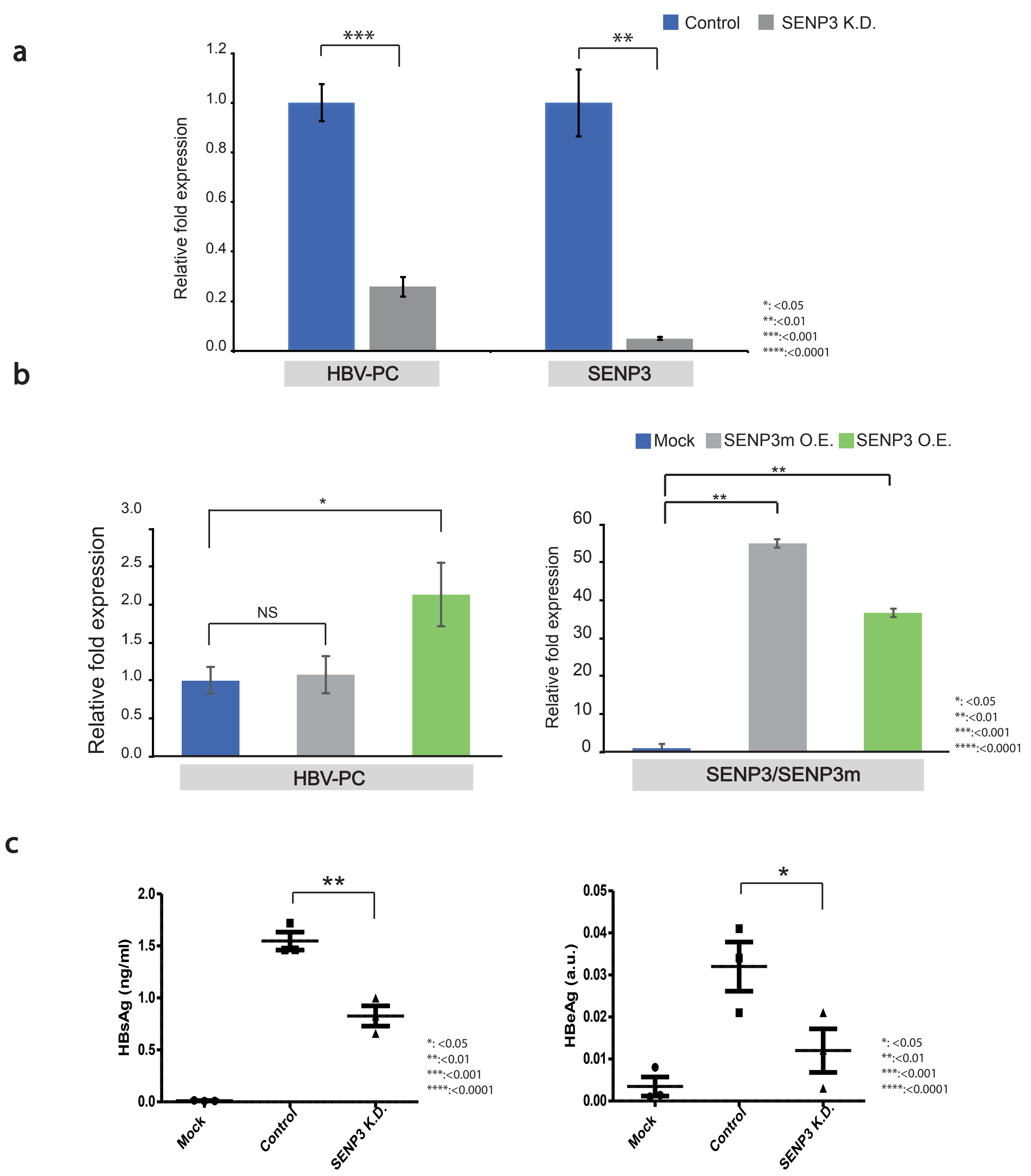

S5 Fig. SENP3 silencing suppresses HBV replication.

(A) RT-qPCR measurements of HBV transcripts amplified by primers HBV-X and HBV-PC in HepAD38-control cells and HepAD38-SENP3 K.D. cells. Beta-actin was used as internal control; the data were expressed as mean±SD (n=3). Statistical significance was assessed by Students' unpaired t-test. (B) Left: RT-qPCR measurement of HBV transcripts amplified by primer HBV-PC after transcient transcription of RGS-SENP3 plasmid and RGS-SENP3m plasmid for ectopic expression of SENP3 or SENP3 mutant (SENP3m) in HepG2.215 cells. Right: RT-qPCR measurement of SENP3 or SENP3m in HepG2.215 cells to indicate the success of ectopic expression. Beta-actin was used as internal control. Beta-actin was used as internal control; the data were expressed as mean±SD (n=3). Statistical significance was assessed by Students' unpaired t-test. (C) Left: HBsAg levels in supernatants of HepG2-NTCP cells (control and SENP3 K.D.) after HBV infection measured by ELISA. Right: HBeAg levels in supernatants of HepG2-NTCP cells (control and SENP3 K.D.) after HBV infection measured by ELISA. Data were mean±SD (n=3). Statistical significance was assessed by Students' unpaired t-test.
