# Supplementary material for: SENP3-mediated host defense response contains HBV replication and restores protein synthesis
Source: PLoS One. 2019 Jan 14;14(1):e0209179. doi: 10.1371/journal.pone.0209179 (PMC6331149; doi:10.1371/journal.pone.0209179)
Supplement: S6 Fig — (A) Immunoblotting of puromycin-labelled proteins in HepG2 and HepG2.215 cells. (B) Immunoblotting of puromycin-labelled proteins in HepG2-control and HepG2-SENP3 K.D. cells. (PDF) [file pone.0209179.s008.pdf]

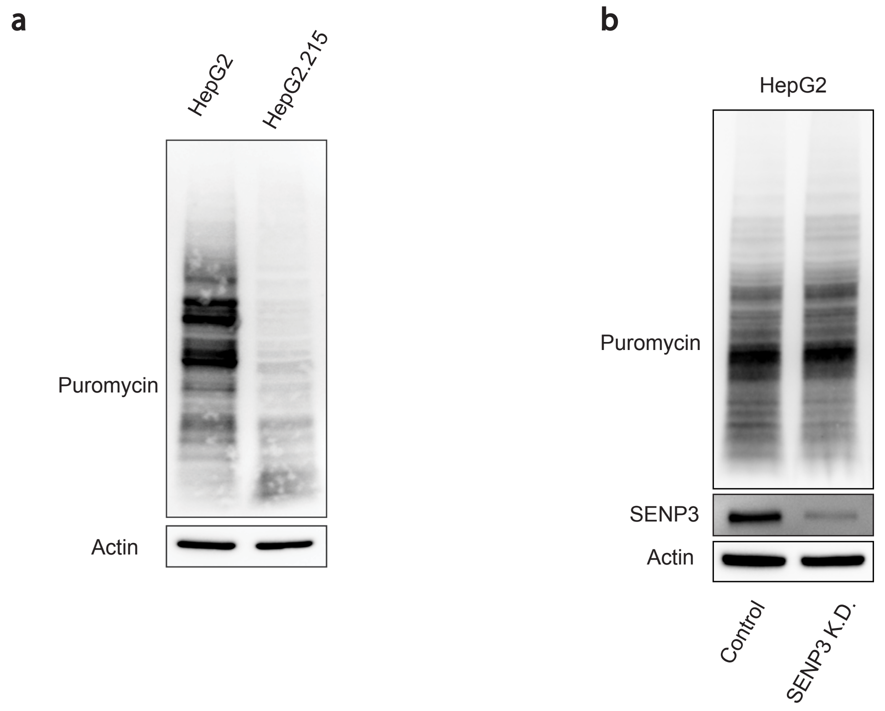

S6 Fig. Translation levels in HepG2 cells.

(A) Immunoblotting of puromycin-labelled proteins in HepG2 and HepG2.215 cells.

(B) Immunoblotting of puromycin-labelled proteins in HepG2-control and HepG2-SENP3 K.D. cells.
