# Supplementary material for: SENP3-mediated host defense response contains HBV replication and restores protein synthesis
Source: PLoS One. 2019 Jan 14;14(1):e0209179. doi: 10.1371/journal.pone.0209179 (PMC6331149; doi:10.1371/journal.pone.0209179)
Supplement: S7 Fig — (A) Quality control of Ribo-seq library from HepG2.215-control cells. (B) Quality control of Ribo-seq library from HepG2.215-SENP3 K.D. cells. (PDF) [file pone.0209179.s009.pdf]

a

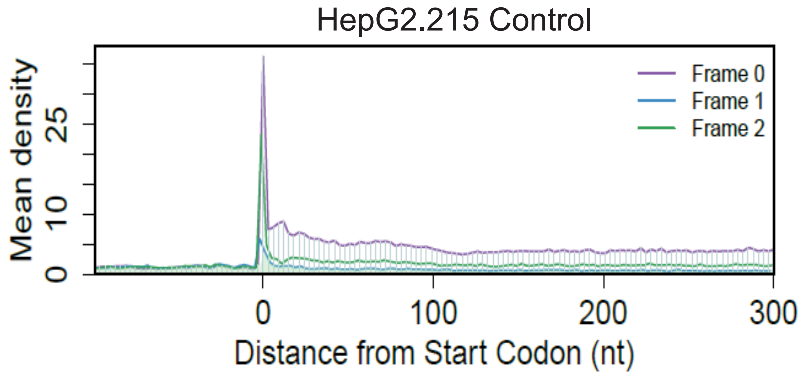

b

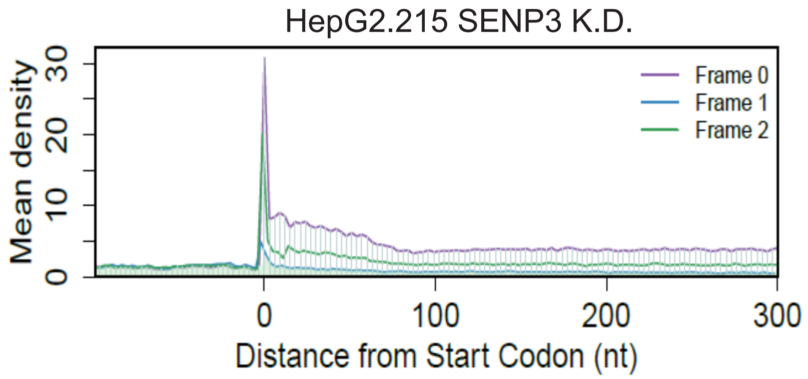

S7 Fig. Ribo-seq quality control.

(A) Quality control of Ribo-seq library from HepG2.215-control cells.

(B) Quality control of Ribo-seq library from HepG2.215-SENP3 K.D. cells.
