# Supplementary material for: SENP3-mediated host defense response contains HBV replication and restores protein synthesis
Source: PLoS One. 2019 Jan 14;14(1):e0209179. doi: 10.1371/journal.pone.0209179 (PMC6331149; doi:10.1371/journal.pone.0209179)
Supplement: S8 Fig — RT-qPCR measurements of HBV transcripts amplified by primers HBV-X and HBV-PC in HepAD38 control cells and IQGAP2K.D. cells. Beta-actin was used as internal control; the data were expressed as mean±SD (n = 3). Statistical significance was assessed by Students’ unpaired t-test. (PDF) [file pone.0209179.s010.pdf]

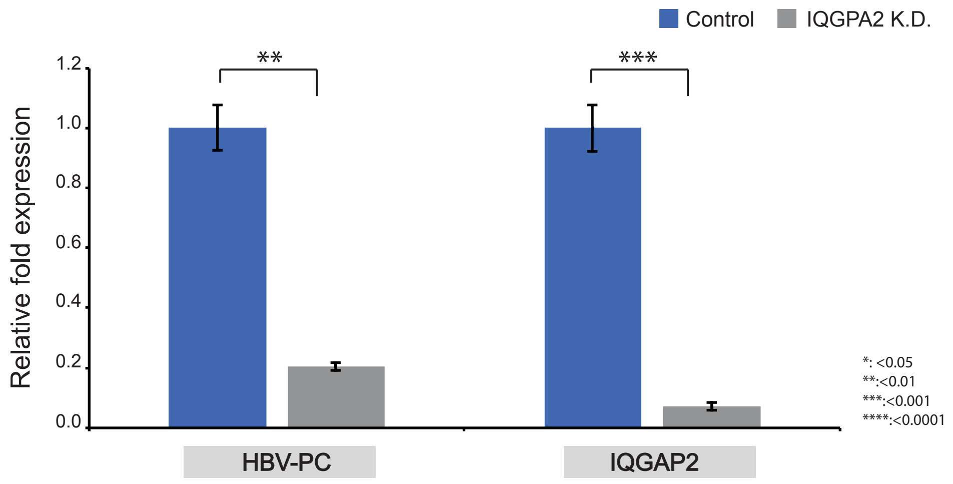

S8 Fig. IQGAP2 silencing suppresses HBV transcription in HepAD38.
RT-qPCR measurements of HBV transcripts amplified by primers HBV-X and HBV-PC in HepAD38 control cells and IQGAP2K.D. cells. Beta-actin was used as internal control; the data were expressed as mean±SD (n=3). Statistical significance was assessed by Students' unpaired t-test.
