# Supplementary material for: SENP3-mediated host defense response contains HBV replication and restores protein synthesis
Source: PLoS One. 2019 Jan 14;14(1):e0209179. doi: 10.1371/journal.pone.0209179 (PMC6331149; doi:10.1371/journal.pone.0209179)
Supplement: S9 Fig — Immunoblotting of SENP3 in HepG2 and HepG2.215 cells after being treated with Rapamycin (20 nM) to inhibit mTOR and LY294002 (20 μM) to inhibit PI3K. Phosphorylated S6 (P-S6) and phosphorylated Akt (p-AKT) were used to indicate inhibition of mTOR and PI3K, respectively. (PDF) [file pone.0209179.s011.pdf]

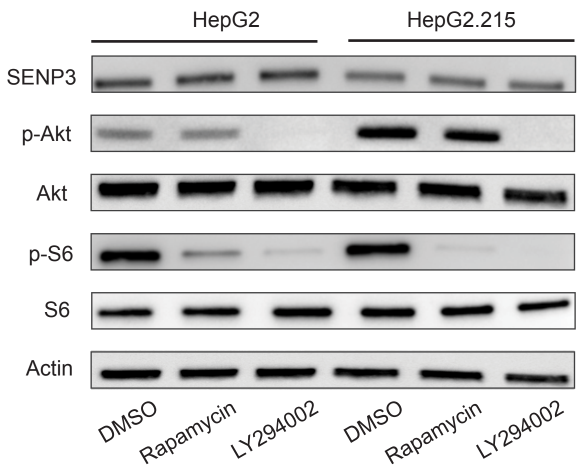

S9 Fig. SENP3 level in HepG2 and HepG2.215 cells after treatment with Rapamycin and LY294002
Immunoblotting of SENP3 in HepG2 and HepG2.215 cells after being treated with Rapamycin (20 nM) to inhibit mTOR and LY294002 (20 μM) to inhibit PI3K. Phosphorylated S6 (P-S6) and phosphorylated Akt (p-AKT) were used to indicate inhibition of mTOR and PI3K, respectively.
